# Limitations of Qdot labelling compared to directly-conjugated probes for single particle tracking of B cell receptor mobility

**DOI:** 10.1038/s41598-017-11563-9

**Published:** 2017-09-12

**Authors:** Libin Abraham, Henry Y. Lu, Rebeca Cardim Falcão, Joshua Scurll, Timothy Jou, Brian Irwin, Reza Tafteh, Michael R. Gold, Daniel Coombs

**Affiliations:** 1 Department of Microbiology & Immunology, University of British Columbia, 2350 Health Sciences Mall, Vancouver, British Columbia V6T 1Z3 Canada; 2 Life Sciences Institute I3 and Cell Research Groups, University of British Columbia, 2350 Health Sciences Mall, Vancouver, British Columbia V6T 1Z3 Canada; 3 Department of Mathematics and Institute of Applied Mathematics, University of British Columbia, 1984 Mathematics Road, Vancouver, British Columbia V6T 1Z2 Canada; 4 Department of Chemistry, University of British Columbia, Vancouver, British Columbia V6T 1Z1 Canada

## Abstract

Single-particle tracking (SPT) is a powerful method for exploring single-molecule dynamics in living cells with nanoscale spatiotemporal resolution. Photostability and bright fluorescence make quantum dots (Qdots) a popular choice for SPT. However, their large size could potentially alter the mobility of the molecule of interest. To test this, we labelled B cell receptors on the surface of B-lymphocytes with monovalent Fab fragments of antibodies that were either linked to Qdots via streptavidin or directly conjugated to the small organic fluorophore Cy3. Imaging of receptor mobility by total internal reflection fluorescence microscopy (TIRFM), followed by quantitative single-molecule diffusion and confinement analysis, definitively showed that Qdots sterically hinder lateral mobility regardless of the substrate to which the cells were adhered. Qdot labelling also drastically altered the frequency with which receptors transitioned between apparent slow- and fast-moving states and reduced the size of apparent confinement zones. Although we show that Qdot-labelled probes can detect large differences in receptor mobility, they fail to resolve subtle differences in lateral diffusion that are readily detectable using Cy3-labelled Fabs. Our findings highlight the utility and limitations of using Qdots for TIRFM and wide-field-based SPT, and have significant implications for interpreting SPT data.

## Introduction

The lateral mobility of plasma membrane receptors is a major determinant of their function and signalling output^1–3^. For many receptors, and especially for activating receptors on immune cells such as T and B cell receptors and Fc receptors, the initiation of receptor signalling is believed to depend on receptor proximity (clustering) as well as the partitioning of positive and negative regulatory molecules into distinct membrane domains^4–7^. Changes in receptor mobility within the membrane may also represent a mode of receptor crosstalk by which one receptor can influence the signalling output of another^8, 9^. Detailed analysis of receptor mobility under multiple conditions can reveal the underlying biophysical mechanisms that shape receptor mobility and organization, and relate these to signalling output and cell activation. Changes in observed mobility over short timescales and distances reflect a heterogeneous membrane environment containing dynamic domains of varying composition, as well as barriers created by the cortical actin cytoskeleton and other cell surface molecules^10–12^. Progressively more detailed spatiotemporal analyses of receptor mobility have generated striking insights into membrane protein dynamics, receptor signalling, and cell activation^13^.

In single-particle tracking (SPT) experiments, the molecule of interest is fluorescently labelled at very low density, allowing individual receptors to be imaged by wide-field, confocal or total internal reflection fluorescence microscopy (TIRFM)^13, 14^. Fluorescent probe selection is of critical importance for SPT as it impacts particle detection, the number and length of tracks obtained, and the assumption that one is imaging single receptor molecules. The two most common labelling strategies for cell surface receptors are: (i) directly conjugating small organic fluorophores to the antigen-binding fragment (Fab) of antibodies, and (ii) conjugating Fab fragments with biotin and then indirectly labelling them with streptavidin (SA)-coupled Quantum dots (Qdots). Other strategies include labelling with micron-sized polystyrene beads. Each of these methods has distinct benefits and drawbacks that may impact the quality and accuracy of the data.

Qdots are semiconductor nanocrystals that allow precise localization due to their bright fluorescence^15^. Moreover, their high photostability allows long tracks to be obtained, thus providing greater insights into phenomena such as directional motion, turning behaviour, state switching, and confinement. This makes them a popular choice for SPT^8, 15–21^. However, Qdot labelling poses certain important concerns^15, 22, 23^. First, there is the potential for steric hindrance and therefore reduced mobility of the receptor-label complex due to its large size (typically ~15–20 nm in diameter). Second, Qdot blinking (occasional switching to a non-fluorescent state) can result in tracking errors. Third, commercially-available SA-conjugated Qdots are intrinsically polyvalent and unless great care is taken, they can potentially bind multiple biotinylated Fab fragments and thus crosslink receptors, altering their motion and potentially initiating signal transduction, causing further changes to receptor mobility.

Directly-labelled monovalent Fab fragments have a simpler stoichiometry and their small size (1–2 nm diameter) reduces the potential for steric hindrance. However, they can exhibit rapid photobleaching (restricting track duration) and they are significantly dimmer than Qdots (reducing tracking precision).

Despite the widespread use of both labelling techniques for SPT, critical side-by-side comparisons of their performance are rare or non-existent in the literature. To help inform fluorophore selection for TIRFM-based SPT, we directly compared these labelling methods in various receptor-tracking experiments and applied multiple analyses to precisely distinguish the results.

The two main steps in SPT, particle detection and track-joining across image frames, have been automated since the early days of SPT, and new methods continue to be developed^24–26^. Once the tracks have been extracted, the simplest analysis is to fit the trajectories to simple Brownian motion. This model has a single parameter, the diffusion coefficient. Tracks obtained under different experimental conditions can then be compared in terms of the fit values for this single parameter^27, 28^. Importantly, methods for determining the diffusion coefficient while accounting for limited positional accuracy have recently been advanced^29–31^. A large number of models extending the Brownian paradigm have also been developed, allowing us to study transient changes in particle behaviour during individual tracks such as fast/slow apparent mobility^32–34^, directed motion^35, 36^, transient binding^16^ and/or anomalous diffusion^37–40^. Similarly, there has been gradual development in our ability to confidently detect zones of transient confinement on the basis of track behavior^41–43^. Although a variety of analysis tools have been developed for SPT, comparisons between studies are limited by the use of different analyses. This highlights the need for a unified, comprehensive, and publicly available analysis platform to streamline the analysis of receptor mobility. We present here a comprehensive workflow and employ multiple recently - developed algorithms to assess single state diffusion, multistate diffusion, and confinement^33, 43^.

By applying these analyses, we directly compare the suitability of Qdot labelling versus Cy3-labelled Fabs for characterizing B cell receptor (BCR) mobility. BCR mobility is a critical determinant of BCR signalling output and ultimately, cell activation. Each naïve mature B cell expresses two forms of the BCR, one containing a transmembrane form of immunoglobulin (Ig)M and the other containing a transmembrane form of IgD. Both the IgM- and IgD-containing BCRs bind the same antigen, and associate with the same intracellular signalling subunit. Why B cells express two different BCRs with identical antigen specificity is a longstanding question. The IgM- and IgD-containing BCRs on a single cell exist in separate nano-domains on the plasma membrane^44^. IgM-BCRs have higher single-state diffusion coefficients than IgD-BCRs, and the two BCR isotypes may be preferentially triggered by monomeric versus multimeric forms of the same antigen^45^. Previously-activated memory B cells express BCR complexes containing a transmembrane form of IgG. These IgG-BCRs have different signalling capabilities and spatial organization^46–48^. However, in all cases, BCR signalling reflects dynamic changes in BCR mobility, proximity and interaction frequency^5^. We show herein that using Cy3-labelled Fabs for TIRFM-based SPT provides greater detection of small differences in BCR mobility than Qdot labelling and avoids the substantial reduction in diffusion rates caused by Qdots.

## Results

### Qdot-labelling hinders cell surface receptor mobility

**Figure 1:**
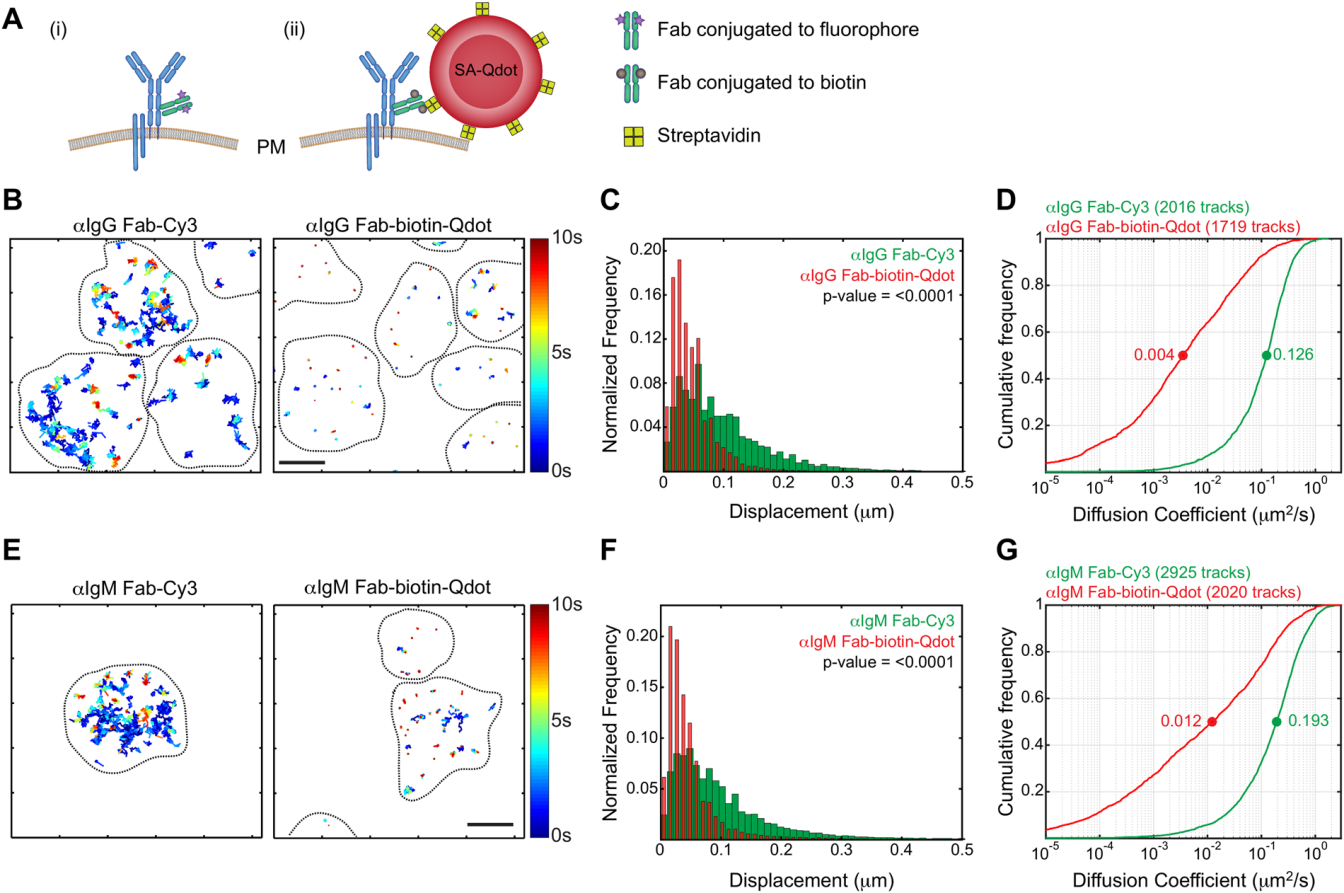
Qdots hinder the lateral mobility of cell surface receptors. (**A**) Schematic representation of BCR labelling for SPT using (i) monovalent Fab fragments directly conjugated to fluorophores, or (ii) monovalent Fab fragments conjugated to biotin, followed by streptavidin (SA)-Qdots. The illustration depicts the relative sizes of the BCR, the Cy3 fluorophore, and SA-Qdot. (**B**,**C**). A20 B-lymphoma cells were labelled with either anti-IgG (αIgG) Fab-Cy3 or anti-IgG Fab-biotin-Qdots, settled onto anti-MHCII-coated coverslips and then imaged for 10 s at 33 Hz by TIRFM. (**B**) Trajectories are plotted using a colour-coded temporal scale. The black dashed lines indicate cell boundaries. (**C**) Distribution of displacement of tracks. Data were analyzed using the Kolmogorov-Smirnov (KS) test and the p-value is indicated. (**D**) Diffusion coefficients were calculated for all tracks and cumulative frequency curves for both labelling strategies are shown. The dots on the curves indicate the median values. (**E**–**G**) *Ex vivo* murine splenic B cells were labelled with anti-IgM (αIgM) Fab-Cy3 or anti-IgM Fab-biotin-Qdots. Trajectories, distributions of displacements, and cumulative frequency curves of diffusion coefficients are shown. Scale bars = 3 μm.

Using TIRFM imaging and SPT to quantify the apparent lateral mobility of IgG-BCRs, we compared results obtained using Fab-biotin-Qdot and Fab-Cy3 labels in parallel experiments (Fig. 1A,B). Mouse A20 B-lymphoma cells were allowed to adhere to coverslips coated with non-stimulatory anti-major histocompatibility complex II (MHCII) monoclonal antibodies^8, 49, 50^ and SPT was performed on the ventral cell surface for 10 s at 33 Hz. Trajectories of individual IgG-BCRs were reconstructed as described in the Methods. Importantly, we confirmed that our labelling protocols predominantly labelled single BCRs on the cell surface (Methods and Supplementary Fig. S1). Although both labelling methods yielded large numbers of tracks that were suitable for analysis, median track lengths using Fab-Cy3 were approximately 30% shorter than with Fab-biotin-Qdot.

The first step in our analysis was to remove particles that were stuck to the cell or coverslip by setting an ‘immobility threshold’ (Methods, Supplementary Video 2; Supplementary Fig. S2). We excluded all tracks that exhibited motion below that of 95% of immobilized fluorescent labels. The proportion of removed tracks never exceeded 2%.

To directly compare the mobility of labelled receptors, we calculated the single-step displacements from all mobile tracks (Methods and Fig. 1C). We found that the choice of label led to significant differences in the apparent lateral mobility of IgG-BCRs, with Qdot labelling yielding substantially (and significantly) lower values.

We next sought to assess the mobility of tracked particles by fitting to the simplest Brownian diffusion model. We confirmed for both labels that the ensemble-averaged mean-square-displacement plot was approximately linear (Supplementary Fig. S3), indicating that this was a reasonable model to consider. To estimate diffusion coefficients from each track, we applied the method of Berglund^29^. This method corrects for two important errors affecting SPT: (i) the limited accuracy of particle position measurements due to diffraction and acquisition errors, especially important at low signal to noise ratio; and (ii) the blurring error induced by long frame acquisition times, where the particle position is effectively averaged over the frame. In order to implement Berglund’s method, we estimated the positional accuracy of our combined imaging and tracking system (Methods). We found a single-frame positional precision of 30 nm for Fab-Cy3 labels and 23 nm for Fab-biotin-Qdot labels. We used these as fixed estimates of positional accuracy while estimating the diffusion coefficients for each track.

We found that the median diffusion coefficient obtained using Fab-biotin-Qdot labels was 35-fold lower than that obtained using Fab-Cy3 labels (Fig. 1D, Supplementary Video 1). A small fraction (10%) of Qdot-labelled receptors displayed high diffusion coefficients (≥0.1 μm^2^/s), but the vast majority exhibited a significantly lower diffusion coefficient than Fab-Cy3-labelled BCRs.

To exclude cell-to-cell variability, simultaneous dual-colour SPT was performed by labelling IgG - BCRs on A20 B cells with limiting amounts of both anti-IgG Fab-Cy3 and anti-IgG Fab-biotin-Qdots (Supplementary Fig. S4). Consistent with the single - label approach, Qdot labels exhibited substantially lower diffusivity than Fab-Cy3 labels on the same cell (Supplementary Fig. S4).

We wished to extend our cell line findings to primary B cells where we could compare the mobility of IgM-BCRs and IgD-BCRs. First, we labelled IgM-containing BCRs on *ex vivo* murine splenic B cells using both anti-IgM Fab-biotin-Qdot and anti-IgM Fab-Cy3. Consistent with our results for IgG on A20 cells, Qdot labels exhibited markedly lower single-step displacements and inferred diffusion coefficients than Fab-Cy3 labels (Fig. 1E–G). Because *ex vivo* primary B cells exhibit minor day-to-day variations in their activation status, we conducted multiple independent experiments on different days, with cells from different mice. Consistently, the median diffusion coefficients obtained using Qdot labels were at least 15-fold lower than those obtained using Fab-Cy3, with only minor variations in the fold difference (Supplementary Fig. S5). Overall, we found that the lateral mobility of cell surface BCRs is significantly reduced when labelled with Qdots and imaged by TIRFM.

Throughout Fig. 1, we show only the Berglund algorithm-generated diffusion coefficients for tracks reflecting measurable receptor mobility. Especially for the Qdot labels, applying the Berglund algorithm results in many trajectories being assigned a very small diffusion coefficient. For example, a diffusing Qdot-labelled particle with D = 10^−4^ μm^2^/s would be expected to travel only (4Dt)^1/2^ = 40 nm during a typical trajectory lasting t = 4 s, well below the Qdot immobility threshold of 180 nm and not far above the Qdot positional accuracy of 21 nm. This result indicates that a fraction of mobile Qdot-labelled receptors are undergoing motion that is not well described by simple diffusion. However, their motion could be accounted for in a model that includes transient changes in mobility between slower- and faster-moving states. We examine this possibility below and also analyze our data using a statistical algorithm that seeks evidence of transient confinement.

### Qdot labelling impairs receptor diffusion on multiple substrates

**Figure 2:**
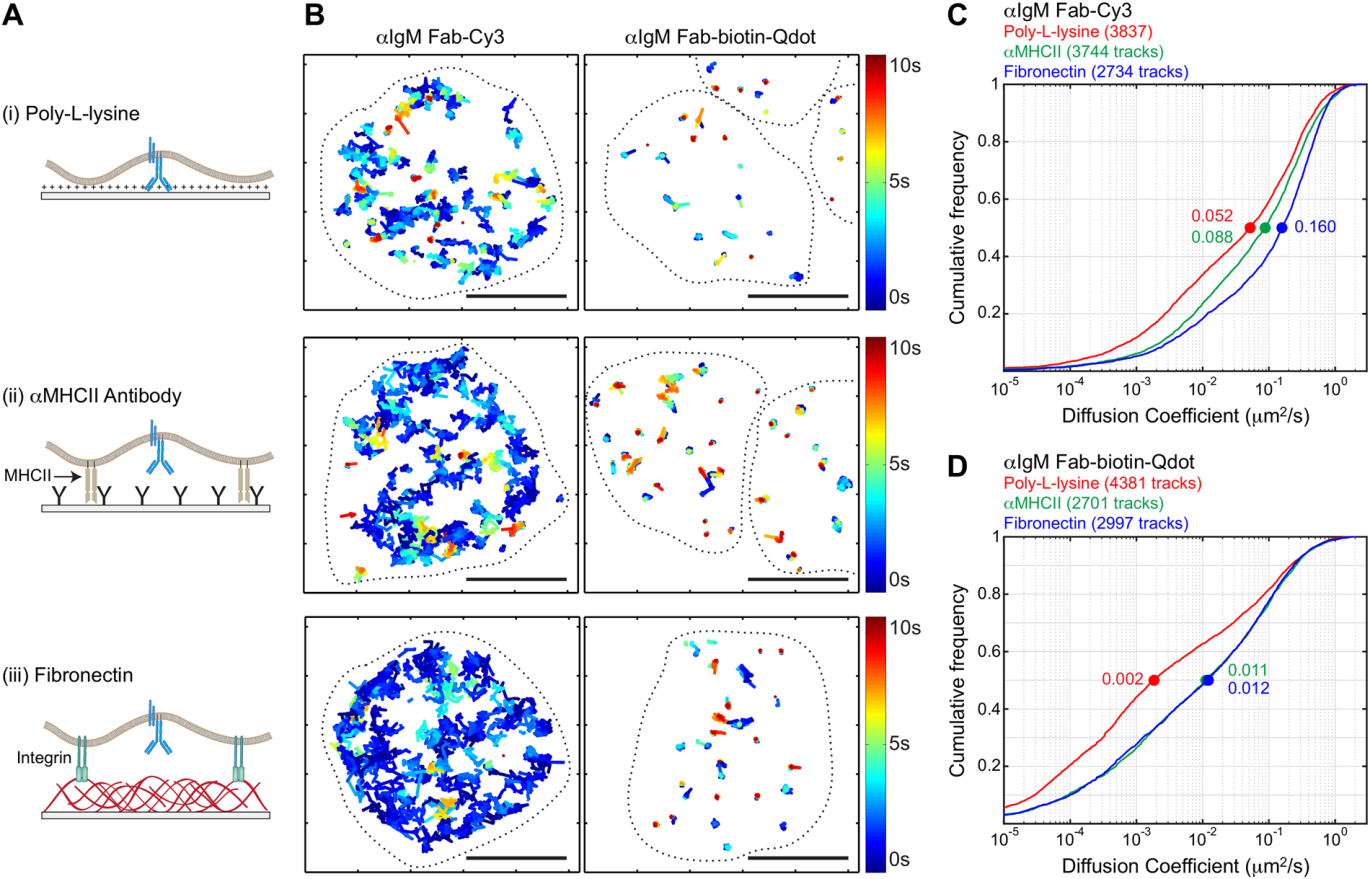
Qdot labelling fails to distinguish substrate-dependent changes in receptor diffusion. (**A**) Schematic representation of coverslips functionalized with (i) poly-L-lysine, (ii) anti-MHCII antibodies (αMHCII), or (iii) fibronectin, indicating the mode of cell attachment and the assumed differences in the distance between the plasma membrane and the coverslip. (**B**) *Ex vivo* murine splenic B cells were labelled and seeded onto the indicated functionalized coverslips (*upper panel* poly-L-lysine, *middle panel* αMHCII, *lower panel* fibronectin) before imaging by TIRFM. Trajectories are plotted using a colour-coded temporal scale. The dashed lines indicate cell boundaries. Scale bars = 5 μm. (**C**,**D**) Single-state diffusion coefficients for IgM-BCR tracks obtained using Fab-Cy3 labelling (**C**) or Fab-biotin-Qdot labelling (**D**). For all three substrates, the cumulative frequency curves of the diffusion coefficients are shown and the median values are indicated by the dots on the curves.

We wished to investigate how the lateral diffusion of labelled receptors depends on the number and strength of adhesive contacts between the cell and the coverslip. Poly-L-lysine, which is commonly used to promote cell adhesion, causes strong, high-valency cellular adhesion via non-specific charge-based interactions. In contrast, non-stimulatory antibodies against cell surface proteins, as well as high molecular weight ligands for cell adhesion receptors (e.g. fibronectin or collagen), generate fewer adhesive contacts. Moreover, because non-stimulatory antibodies and extracellular matrix proteins bind to cell surface receptors, as opposed to the plasma membrane, they may allow more space between the cell and coverslip (Fig. 2A).

Primary B cells were plated on coverslips that had been functionalized with poly-L-lysine, non-stimulatory anti-MHCII antibodies, or fibronectin. Consistent with our hypothesis that close juxtaposition of the cells to the coverslip would reduce receptor mobility, we found that IgM-BCR diffusion was slowest when the cells were plated on poly-L-lysine-coated coverslips (Fig. 2B, *upper panel*, Fig. 2C,D), compared to coverslips coated with anti-MHCII or fibronectin (Fig. 2B, *middle and lower panel*, Fig. 2C,D). This was true regardless of labelling strategy. However, as in Fig. 1, the median diffusion coefficients for Qdot-labelled receptors were 8- to 28-fold lower than for Fab-Cy3-labelled receptors (Fig. 2C,D), for all substrates.

Importantly, we found that Fab-Cy3 labelling provided superior detection of subtle differences in receptor mobility. Fab-Cy3 labelling indicated an IgM-BCR median diffusion coefficient that was ~1.8-fold higher for cells plated on fibronectin versus anti-MHCII (Fig. 2C,D). Although Qdot-labelled BCRs were slowest on poly-L-lysine, we could not discern differences in mobility between cells plated on anti-MHCII versus fibronectin. We conclude that Fab-Cy3 labelling enables superior discrimination of small differences in receptor mobility.

### Qdot labelling substantially alters receptor behaviour as assessed by a multistate diffusion model

The lateral mobility of molecules within the plasma membrane is influenced by interactions with the cortical actin cytoskeleton or with membrane compartments of distinct compositions and viscosity such as lipid rafts, protein islands, and tetraspanin-enriched microdomains^13, 49, 50^. Therefore, a receptor may transiently move faster or slower as it explores its environment. We have previously developed a simple two-state model that assumes that receptor trajectories can be broken down into two distinct diffusive modes, each characterized by a single diffusion coefficient^33^. Transitions between the two states are assumed to occur according to a Poisson (memoryless) process with two fixed transition rates. This forms a four-parameter Hidden Markov Model (HMM) model, in contrast to the single parameter of a pure diffusion model. We can obtain the maximum likelihood values of the parameters for a given set of tracks, and investigate how different experimental conditions affect the frequency of switching between fast- and slow-moving states, as well as the diffusion coefficients associated with each state^33^. Specifically, we wished to determine whether the fast → slow and slow → fast switching rates obtained using Fab-Cy3 and Fab-biotin-Qdot labelling were similar, even though the overall mobility of Qdot-labelled receptors was reduced compared to Fab-Cy3-labelled receptors.

**Figure 3:**
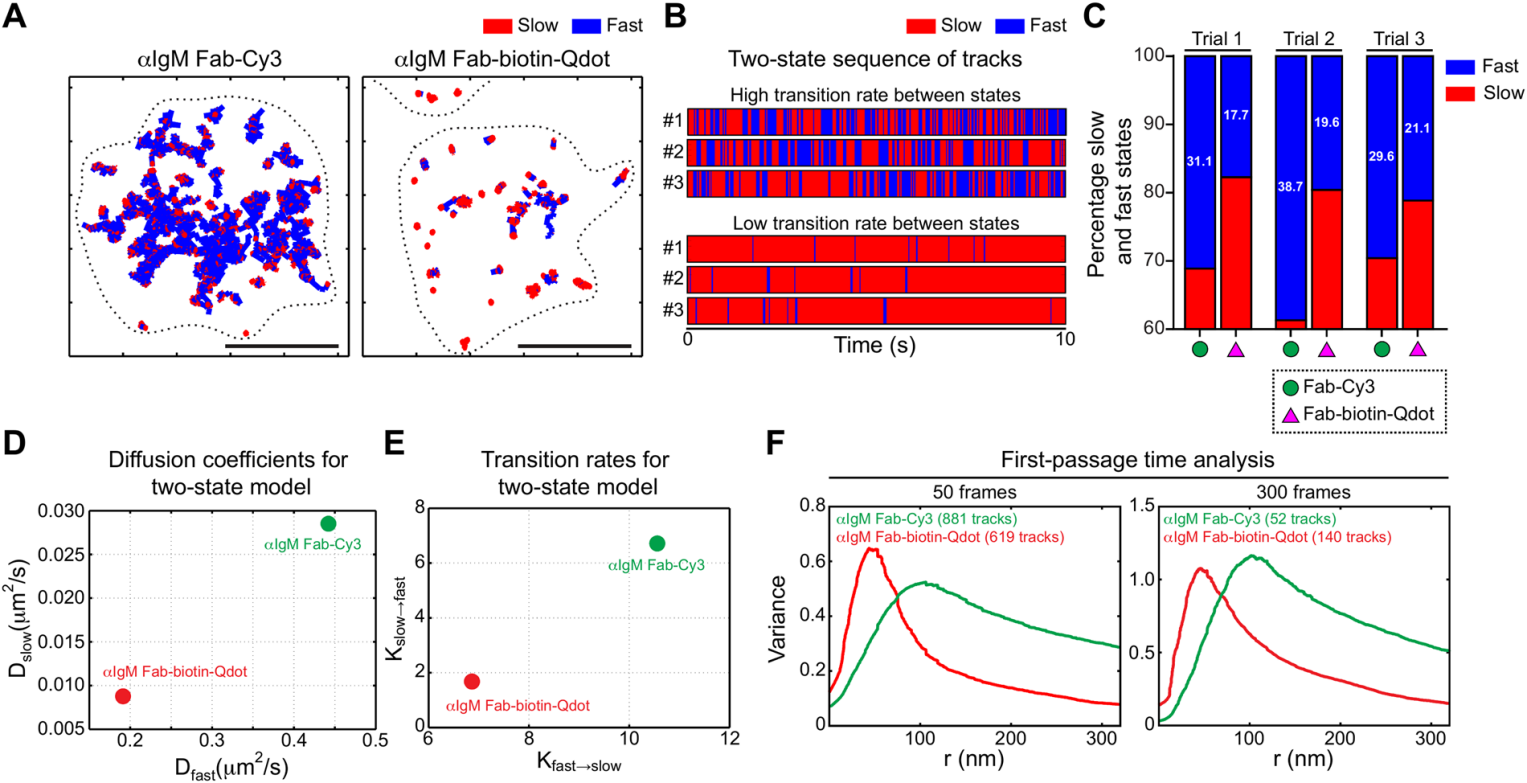
Qdot labelling alters state-switching behaviour and apparent confinement of IgM BCRs. *Ex vivo* murine splenic B cells were labelled with anti-IgM Fab-Cy3 or anti-IgM Fab-biotin-Qdot and adhered to anti-MHCII-coated coverslips before being imaged for 10 s at 33 Hz. (**A**) After applying an immobility threshold to remove stuck particles (see Methods and Supplementary Fig. S2), a two-state HMM model was used to subdivide trajectories into slow-diffusing (red) and fast-diffusing (blue) segments, with dynamic transitions between these two inferred behaviours. Depicted are representative static trajectories of IgM-BCRs that were segmented into inferred slow and fast states. Scale bar = 5 μm. (**B**) Each barcode shows the time course for transitions between fast (blue) and slow (red) states. Shown are 3 examples of trajectories in which the receptor rapidly switches between slow and fast states (these were obtained using anti-IgM Fab-Cy3 labelling) with a high transition rate and 3 trajectories in which the receptor exhibits primarily slow diffusion, with a low transition rate (these were obtained using anti-IgM Fab-biotin-Qdot labelling). (**C**) For each condition, all tracks were combined and the percent of time that receptors exhibited slow (red) versus fast (blue) diffusion was determined. Data are shown for 3 independent experiments. (**D**,**E**) The HMM algorithm was then used to calculate the inferred slow and fast state diffusion coefficients (**D**), as well as the transition rates (K_slow→fast_, K_fast→slow_) (**E**) between the two states for receptors labelled with either anti-IgM Fab-Cy3 (green) or anti-IgM Fab-biotin-Qdot (red). Note the lower values for Fab-biotin-Qdot labelling. (**F**) After applying an immobility threshold to remove stuck particles, the trajectories were analyzed using the first-passage time algorithm. Histograms depicting the confinement radii for short tracks (50 frames, *left panel*) and long tracks (300 frames, *right panel*) are shown. The numbers of tracks analyzed are indicated. Note that Fab-Cy3 labelling yields larger confinement radii.

We applied our two-state HMM analysis^33^ to compare the impact of Fab-Cy3 versus Fab-biotin-Qdot labelling on inferred fast/slow state switching. IgM-BCRs on *ex vivo* splenic B cells were imaged by TIRFM and we fit the two-state HMM independently for each experimental condition. We found that receptors labelled with Fab-Cy3 were more likely to be found in a fast-diffusing state compared to Qdot-labelled receptors (Fig. 3A–D), and that both the fast- and slow-diffusing states showed higher diffusivity when Fab-Cy3 labelling was used. Importantly, Fab-Cy3-labelled BCRs exhibited higher state transition rates than Qdot-labelled receptors, which were mainly restricted to a slow-moving state (Fig. 3B,C,E). These findings were recapitulated in multiple independent experiments (Supplementary Table S1) and similar results were obtained for IgG - BCRs on A20 cells (Supplementary Fig. S6).

Overall, this analysis provides a refined picture of the relative effects of the different labelling strategies. Qdot labelling reduces diffusivity in both the fast and slow states, and additionally impacts the transition rates, with the result that Qdot-labelled receptors are predominantly inferred to be in the slow state.

### First-passage-time analysis reveals that Qdot-labelled receptors exhibit substantial apparent confinement

Physical barriers created by the submembrane cytoskeleton or large transmembrane proteins are believed to restrict the lateral mobility of receptors in the plasma membrane^10, 13^. Such barriers are dynamic and result in transient receptor confinement, which impacts receptor interactions and signalling. Hence, there is considerable interest in determining the sizes of apparent confinement zones and investigating cellular stimuli that alter such confinement zones.

To assess track confinement, we applied a modified first passage time (FPT) analysis^43^ (also see Methods). The FPT analysis was first tested on simulated data using parameters originally described by Rajani *et al*.^43^ (Supplementary Fig. S7). We further modified and tested this algorithm on simulated data with parameters similar to our experimental data, in order to verify how the algorithm accommodates tracks of different lengths. Because the FPT analysis is influenced by track length (Methods and Supplementary Fig. S7), we compared tracks of the same length (Fig. 3F). For both IgM-BCRs on splenic B cells and IgG-BCRs on A20 cells, we found that Qdot labelling consistently yielded smaller effective confinement radii (radii corresponding to peak variance in escape time), compared to Fab-Cy3 labelling (Fig. 3F, Supplementary Fig. S6). This aligns with the results obtained using the two-state HMM model, which showed that Qdot-labelled receptors exhibited decreased state-switching behaviour, a greater fraction of the receptors in the slow state, and reduced diffusion coefficients for both states compared to Fab-Cy3-labelled receptors. Taken together, these results show that Qdot-labelled BCRs exhibit reduced mobility relative to Fab-Cy3-labelled BCRs, and that they are apparently restricted to smaller effective confinement regions.

### Qdot labelling obscures subtle differences in the diffusion kinetics of different receptors

**Figure 4:**
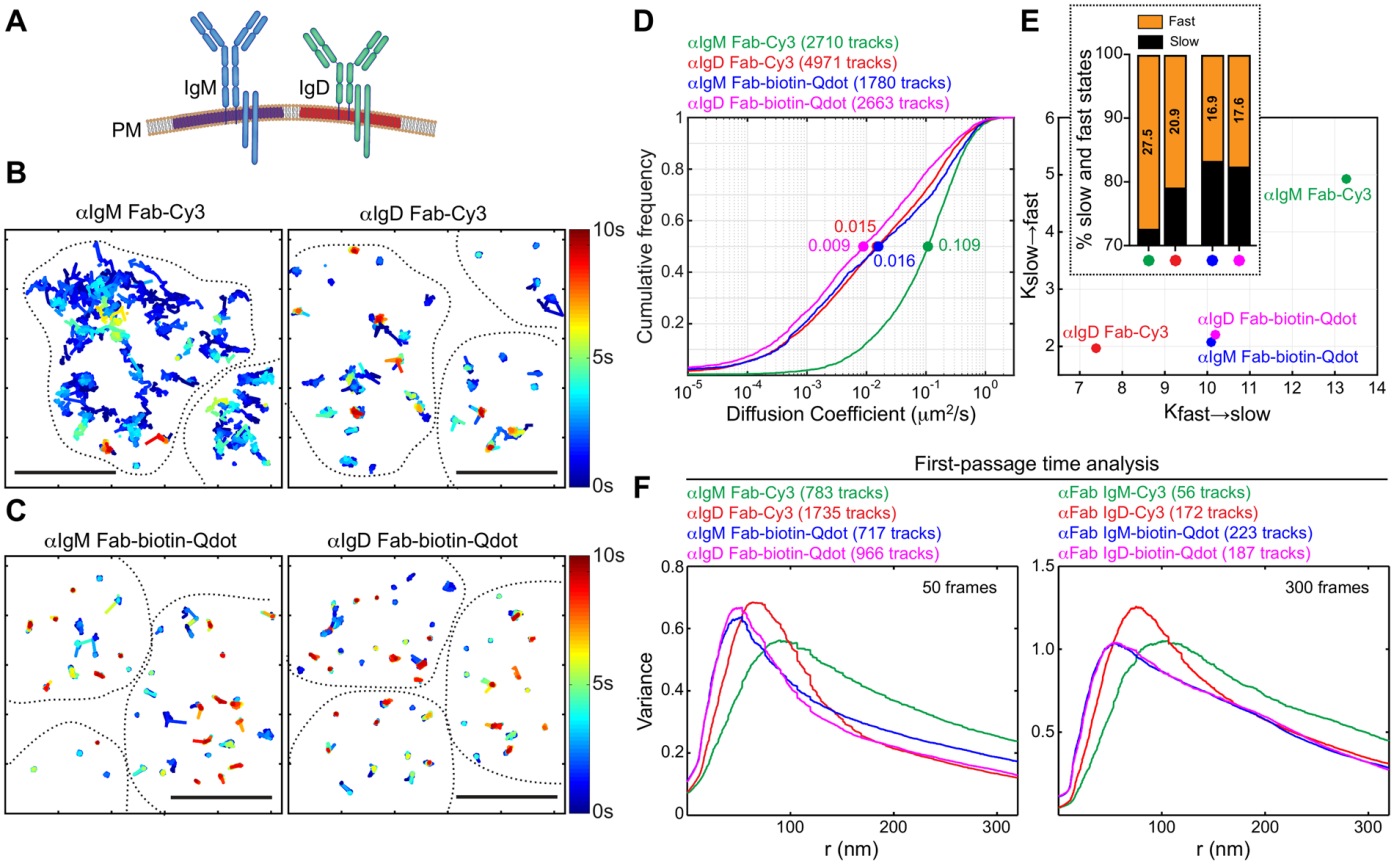
Lateral diffusion of IgM-BCRs and IgD-BCRs cannot be distinguished using Qdot-labelling. (**A**) Schematic representation of IgM- and IgD-containing BCRs. (**B**–**F**) *Ex vivo* splenic B cells were labelled with anti-IgM or anti-IgD (αIgD) Fab-Cy3 probes (**B**) or with anti-IgM or anti-IgD Fab-biotin-Qdot probes (**C**). In panels B and C, trajectories are plotted using a colour-coded temporal scale. The dashed lines indicate cell boundaries. Scale bars = 5 μm. Cumulative frequency plots of single-state diffusion coefficients are shown (**D**). Median values are indicated by the dots. In panel (**E**), tracks were analyzed using the FPT algorithm. Histograms depicting the confinement radii for short tracks (50 frames, *left panel*) and long tracks (300 frames, *right panel*) are shown. In panel (**F**), the tracks were analyzed using the two-state HMM. The transition rates (K_slow→fast_, K_fast→slow_) between the two states are shown. The inset shows the fraction of time that receptors exhibited slow (red) versus fast (blue) diffusion, as determined using the two-state HMM. All data in panels (**B**–**F**) are from the same experiment. Similar results were obtained in two independent experiments.

Mature primary B cells express both IgM- and IgD-containing BCRs that recognize the same antigen (Fig. 4A). IgM- and IgD-containing BCRs exist in separate protein islands and IgD-BCRs exhibit slower lateral diffusion than IgM-BCRs^44, 49^. Although these differences in receptor diffusivity are fairly small, they may have important consequences for BCR signalling. Hence, we compared the ability of the different labelling strategies to detect these differences.

First, we used Fab-Cy3 labelling to assess the mobility of IgM and IgD in separate experiments (Fig. 4B,C). As previously shown^49^, the lateral mobility of cell surface IgM-BCRs, as characterized by median single-state diffusion coefficient, was ~7-fold higher than that for IgD-BCRs (Fig. 4D, red *vs*. green). We then used the HMM two-state diffusion model described above to estimate transition rates between inferred fast and slow states (Fig. 4E, Supplementary Table S2). IgM and IgD were both found to spend more time in the inferred slow state than the fast state (Fig. 4E, inset). However, transition rates associated with IgM were substantially higher, perhaps because IgM more rapidly enters and exits regions of decreased mobility, or has frequent but transient binding interactions with a comparatively immobile partner. We then used the FPT algorithm and found that IgM exhibited a slightly larger confinement radius than IgD (Fig. 4F, red *vs*. green).

In sharp contrast, labelling IgM and IgD with Fab-biotin-Qdots yielded tracks that showed only subtle differences in diffusivity (Fig. 4D, blue *vs*. magenta), and which were virtually indistinguishable in terms of transition rates (Fig. 4E, Supplementary Table S2) and confinement radii (Fig. 4F, blue *vs* magenta). All of these results were independently confirmed using different mice (Supplementary Fig. S8). These results definitively show that Qdot labelling fails to detect subtle differences in receptor mobility in SPT-TIRFM experiments.

### Qdot labelling can be used to detect larger differences in receptor mobility

Since Qdots have been chosen for many SPT studies because of their brightness and high signal-to-noise ratio^15, 51, 52^, we asked whether Qdot labelling was a suitable tool for detecting large changes in receptor mobility.

**Figure 5:**
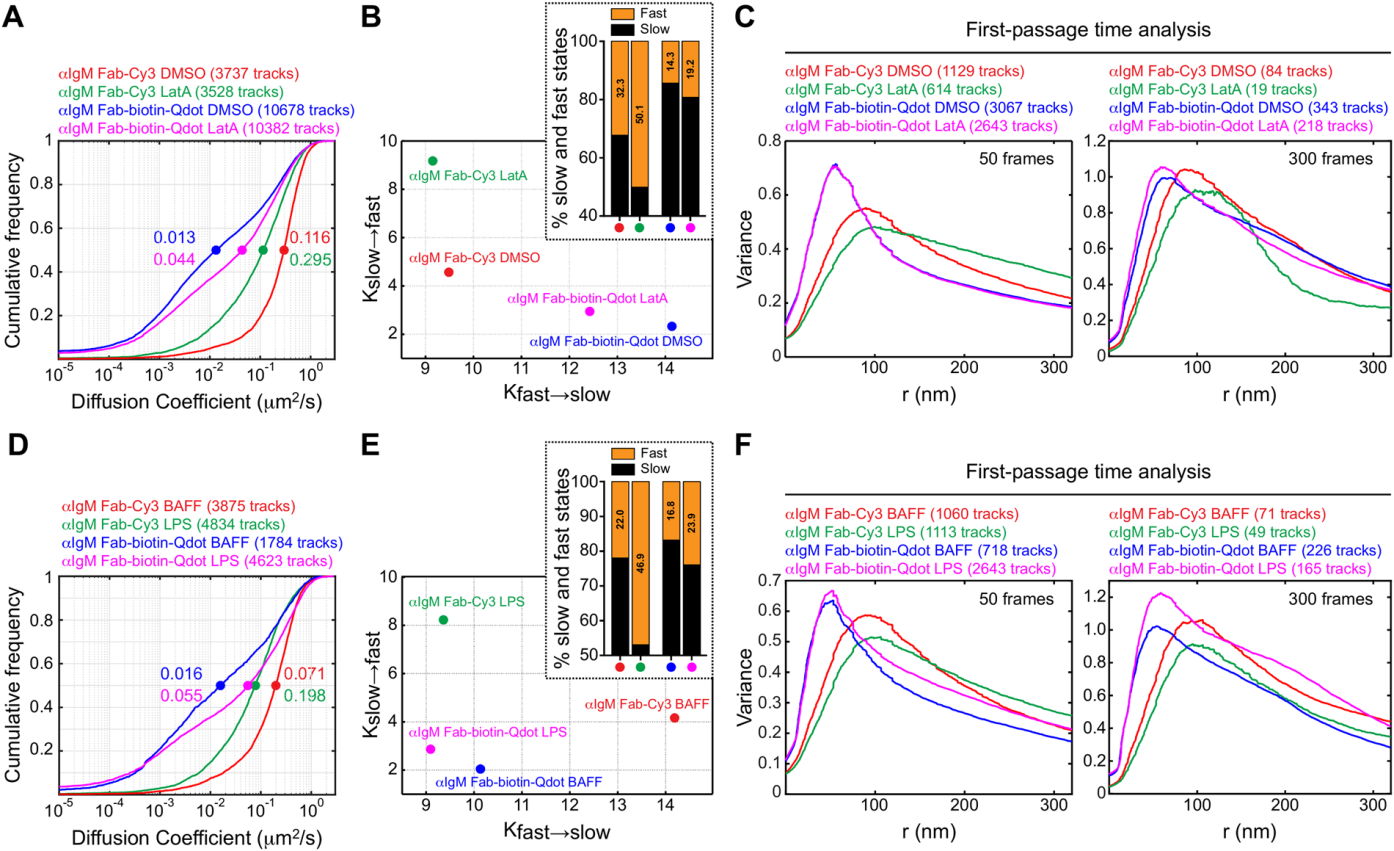
Larger changes in receptor mobility can be detected using Qdots. (**A**–**C**) *Ex vivo* splenic B cells were labelled with either anti-IgM Fab-Cy3 or anti-IgM Fab-biotin-Qdot. The cells were treated with DMSO or 1 μM latrunculin A (LatA) for 5 min prior to SPT imaging. Cumulative frequency plots of single-state diffusion coefficients for IgM-BCRs are shown in (**A**). Median values are indicated by the dots. (**B**) The tracks were analyzed using the two-state HMM. The transition rates (K_slow→fast_, K_fast→slow_) between the two states are shown. The inset shows the fraction of time that receptors exhibited slow (black) versus fast (orange) diffusion, as determined using the two-state HMM. All data are from the same experiment. Similar results were obtained in three independent experiments. (**C**) The trajectories were analyzed using the FPT algorithm and histograms depicting the confinement radii for short tracks (50 frames, *left panel*) and long tracks (300 frames, *right panel*) are shown. (**D**–**F**) Splenic B cells were cultured for 16 h with 5 ng/mL BAFF or with BAFF + 5 μg/mL LPS before being labelled with either anti-IgM Fab-Cy3 or anti-IgM Fab-biotin-Qdot. (**D**) Cumulative frequency plots of diffusion coefficients are shown. (**E**) The transition rates between slow- and fast-diffusion states determined by the HMM algorithm. The fraction of time that receptors exhibited slow (black) versus fast (orange) diffusion are shown in the inset. (**F**) The trajectories were analyzed using the FPT algorithm and histograms depicting the confinement radii for short and long tracks are shown. All data are from the same experiment. Similar results were obtained in two independent experiments.

We compared the ability of Qdot and Fab-Cy3 labelling to detect changes in BCR mobility that occur when the submembrane actin cytoskeleton is disrupted by latrunculin A (LatA). In agreement with our previous work^8^, we found that LatA treatment increased the diffusivity of IgM-BCRs (Fig. 5A). Importantly, this effect was detected equally well by both labelling strategies. Although receptors labelled with Qdots generally had lower diffusion coefficients than those labelled directly with Fab-Cy3, the resulting fold increases in receptor mobility caused by LatA treatment were quite similar. The two-state diffusion model showed that the rate of transitioning from slow-to-fast (K_slow__→__fast_), as well as the proportion of time spent in the fast state, was higher after LatA treatment, regardless of the labelling approach, although these differences were much less pronounced when Qdots were used (Fig. 5B, Supplementary Table S2). Applying the FPT algorithm, we found that LatA treatment slightly increased the apparent confinement radii of receptors labelled using Fab-Cy3, consistent with previous reports^8, 49^ (Fig. 5C), although this was not a strong effect. However, BCRs labelled with Qdots showed almost no change in apparent confinement radii after LatA treatment and appeared to remain tightly confined. Hence, Qdot-labelling allowed us to distinguish diffusion coefficients and state-switching behaviour but not small changes in confinement radii that were detectable using Fab-Cy3 labelling.

We have also previously shown that treating primary B cells with Toll-like receptor (TLR) ligands increases the diffusivity of IgM-BCRs and decreases IgM confinement by activating the actin-severing protein cofilin^8^. As a further test, we compared the ability of the two labelling strategies to detect these changes. We confirmed that treating primary B cells with the TLR ligand lipopolysaccharide (LPS) induced a robust (~2.5-fold) increase in the single-state diffusion coefficient for IgM-BCRs (Fig. 5D). Although the diffusion coefficients obtained via Qdot-labelling were lower than their Fab-Cy3-labelled counterparts, the fold-increase after LPS treatment was similar for both labelling strategies. Both methods showed that LPS treatment increases the K_slow__→__fast_ transition rate and the proportion of IgM - BCRs in the inferred fast state. However these differences were much less evident with Fab-biotin-Qdot labelling (Fig. 5E, Supplementary Table S2). Minor changes in confinement radius following LPS treatment were also detected using both labels (Fig. 5F).

Finally, to determine whether the decreased mobility of Qdot-labelled receptors was a consequence of steric hindrance at the cell-coverslip interface, we directly compared the motion of BCRs on the dorsal side of cells with their counterparts at the cell-coverslip interface (Methods, Supplementary Fig. S9, Supplementary Table S3). We found that Fab-biotin-Qdot - labelled BCRs imaged on the dorsal side of cells by wide-field imaging showed slightly higher mobility, compared to comparably labelled BCRs imaged by TIRFM. However, they were still substantially less mobile than BCRs labelled with Fab-Cy3 and imaged by TIRFM. We conclude that Fab-biotin-Qdot labelling may impose substantial restrictions on receptor mobility even away from the potentially restrictive cell-coverslip interface.

### A multiparameter analysis workflow for single particle tracking experiments

Our results depend heavily on computational analysis of the experimental tracks. In order to facilitate consistent analysis among different groups and reports, we have developed a MATLAB-based graphical user interface (GUI) called SPT-TIRF Track Analyzer for all of the analyses presented here (available on request).

## Discussion

Particle - tracking experiments can provide valuable insights into how the dynamics of cell surface receptors are regulated and how receptor mobility influences signalling output. However, there are few comparative studies of labelling strategies for SPT and how they affect the interpretation of SPT data. We have conducted a comprehensive multi-parameter analysis of BCR mobility using Fab-Cy3 and Fab-biotin-Qdot labelling, allowing us to provide a detailed critical comparison. We quantified the effects of labelling by subjecting our data to simple diffusion coefficient estimation, a multistate HMM diffusion model, and an FPT algorithm for determining the apparent confinement radius of a track. We consistently found that Qdot-labelled receptors exhibit reduced mobility, lower transition rates between inferred diffusive states, and a higher degree of apparent confinement, in both TIRFM and wide-field imaging. Although large changes in receptor mobility were qualitatively detected by Qdot-labelling, Fab-Cy3 labelling provides superior quantification of receptor mobility. Based on our findings, we strongly advise against using Fab-biotin-Qdot constructs for labelling receptors on B cells. Whether Qdot-labelling is appropriate for use in other situations can only be determined via careful controls using alternative labelling approaches such as directly-conjugated Fab fragments^14, 53–57^.

There are several possible reasons for the reduced mobility of Qdot-labelled receptors. The first reason relates to the potential for receptor crosslinking. We minimized this possibility by using limiting dilutions of Fabs and Qdots, but although it is possible to engineer monovalent Qdots with a single bound Fab^16, 18, 58^, monovalency is a significant benefit of directly-labelled Fab fragments. Qdots can also have cytotoxic effects that may influence the physiology of the cell and hence the mobility of the receptor^59, 60^.

Steric hindrance of motion at the cell-substrate interface is another potential reason for the reduced mobility of receptors labelled with Qdots or other bulky probes such as polystyrene beads. Piehler and colleagues showed that specific combinations of Qdot size and substrate can reduce substrate interactions, but comparisons with other labelling modalities were not described^61^. We find that different substrates appear to hinder Qdot (and, to a lesser extent, Fab-Cy3) motion to degrees correlating approximately with the thickness of the molecular layer of adhesion molecules (Fig. 2). Small streptavidin-conjugated Qdots (~9 nm in diameter) have been developed and should be much less prone to steric hindrance^62^. However, these are not yet commercially available and are reported to suffer from poor photostability^63^. Steric hindrance would presumably be much reduced by imaging on the dorsal (top) side of cells. Indeed, a previous study reported that the mobility of Fcγ receptors on primary macrophages was essentially equivalent using Fab-Cy3 and Qdot labels via dorsal imaging^17^. Here, we found that dorsal imaging of Qdot labels slightly increased apparent mobility, but it was still lower than that of Fab-Cy3 labels imaged at the cell-substrate interface. We note that dorsal imaging ideally requires a flat dorsal membrane, as seen predominantly in macrophages^17^ and is technically challenging for round cells such as lymphocytes.

A drawback of labelling with organic fluorophores is their lower brightness, leading to reduced resolution in tracking, and their susceptibility to photobleaching. Qdots, in contrast, do not bleach but can suffer from blinking. This can be partially suppressed by reducing agents, or by using carefully-engineered Qdots^63^. Here, we have shown that Fab-Cy3 labelling allows reliable imaging and tracking at 33 Hz and that we can obtain a large number of sufficiently long tracks to perform our analyses. However, if very long tracks are essential, Qdot labelling may be required. In that case, tracking must be performed carefully so that blinking events are dealt with properly.

Interpreting SPT data requires a well-defined mathematical process. Here, we provide an integrated track analysis platform that takes output from a track-processing program^24, 64, 65^. As well as providing the diffusion coefficient for a track, it allows the user to estimate apparent confinement via FPT analysis and use an HMM to detect dynamic changes in diffusivity^33^. Our platform provides a user-friendly entrée to SPT analysis and may help standardize analysis approaches.

Focusing on Fab-Cy3 labelling, our estimated diffusion coefficients for IgM- and IgD-BCRs on primary B cells, and for IgG-BCRs on A20 cells, agree with published results^49, 50^. By applying the two-state and FPT analyses, we gained additional insights into how the mobility of IgM-BCR and IgD-BCR differ. We found that IgM-BCRs cycle more rapidly between the apparent fast- and slow-states than IgD-BCRs, and spend more time in the fast state. Moreover, FPT analysis reveals that IgD-BCRs are more tightly confined with smaller apparent confinement zones than IgM-BCRs. IgM-BCRs and IgD-BCRs exist in distinct nanoscale membrane domains and this may account for the differences in their mobility^44, 49^. Because BCR organization and mobility strongly influence BCR signalling^5, 9, 44, 50^, detailed analysis will provide insights into the differential regulation of signalling by IgM- and IgD-BCRs.

In summary, we have presented a comprehensive analysis of BCR motion at a cell-substrate interface using TIRFM-based SPT. We find substantially different results depending on labelling modality and recommend extreme caution when using Qdot labelling. We have emphasized the role of mathematical analysis in interpreting SPT and to that end have produced a tracking analysis platform and GUI. Additionally, in keeping with the principle of reproducible research and to support the development of new methods for track extraction and SPT analysis, we will supply all imaging and tracking data upon request.

## Materials and Methods

### B cell isolation and culture

Splenic B cells were obtained from 6- to 10-week old C57BL/6 mice of either sex (Jackson Laboratories) using a B cell isolation kit (STEMCELL Technologies; catalogue #19854A) to deplete non-B cells. The University of British Columbia Animal Care Committee approved all animal protocols and all animal experiments were carried out in accordance with institutional regulations. A20 B-lymphoma cells were obtained from ATCC. B cells were cultured in RPMI-1640 (Life Technologies; catalogue #21870-076) supplemented with 10% heat inactivated fetal bovine serum, 2 mM L-glutamine, 50 μM β-mercaptoethanol, 1 mM sodium pyruvate, 50 U/mL penicillin, and 50 μg/mL streptomycin (complete medium). Where indicated, splenic B cells were cultured for 16 h with recombinant murine B cell activating factor (BAFF) (R&D Systems; catalogue #2106-BF-010) with or without *E. coli* 0111:B4 LPS (Sigma-Aldrich; catalogue #L2630).

### Functionalization of glass coverslips

Before use, acid-cleaned glass coverslips (Marienfeld #1.5H, 18 × 18 mm; catalogue #0107032, Lauda-Königshofen, Germany) were incubated with 0.01% poly-L-lysine (Sigma-Aldrich; catalogue #P4707), 0.25 µg/cm^2^ of the M5/114 anti-MHCII monoclonal antibody (Millipore; catalogue #MABF33), or 1 µg/cm^2^ fibronectin (Sigma; catalogue #F4759-5MG) for at least 3 h at 37 °C. The slides were then washed with phosphate-buffered saline (PBS) and blocked with 3% bovine serum albumin.

### Monovalent Fab fragments

The following Fab fragments were obtained from Jackson ImmunoResearch Laboratories (West Grove, PA): Cy3-conjugated anti-IgM Fab (catalogue #115-167-020), biotinylated anti-IgM Fab (catalogue #115-067-020), Cy3-conjugated anti-IgG Fab (catalogue #115-167-003), and biotinylated anti-IgG Fab (catalogue #115-067-003). The anti-mouse IgD monoclonal antibody was isolated from hybridoma supernatants (clone 11-26c) and Fab fragments from this antibody were prepared by AbLab (Vancouver, Canada) using a Fab Preparation Kit (Pierce; catalogue #44985). Fab fragments were separated from Fc fragments using an anti-mouse IgG Fc-specific Sepharose column. Isolated Fab fragments were concentrated and exchanged into buffers suitable for Cy3 or biotin conjugation using an Amicon Ultra 3 K spin concentrator (Millipore). All Fab fragments were routinely tested for aggregation using dynamic light scattering (Zetasizer Nano) and only Fab preparations with unimodal size distribution were used for experiments.

### Receptor labelling for single particle tracking

Receptor labelling for SPT was performed as previously described^8, 49^. All steps were done at 4 °C in order to minimize BCR clustering and endocytosis. Briefly, *ex vivo* splenic B cells or A20 cells were resuspended to 10^7^ cells/mL in ice-cold PBS, blocked with 5% rat serum for 10 min and then labelled for 5 min with limiting dilutions of Fab fragments. The final concentrations used were 1 ng/mL Cy3- or biotinylated anti-IgM Fab fragments, 100 ng/mL Cy3- or biotinylated anti-IgD Fab fragments, and 1 ng/mL Cy3- or biotinylated anti-IgG Fab fragments. The average fluorophore to protein ratio for the Cy3-labelled Fab fragments was 2.3. When biotinylated Fab fragments were used, the cells were subsequently incubated for 1 min with streptavidin-conjugated 525 nm Qdots (Invitrogen; catalogue #Q10143MP; 1:1000 dilution of stock suspension, 1 nM final concentration). After labelling, the cells were washed twice with RPMI-1640 as a source of free biotin to prevent streptavidin-mediated clustering of the BCR. The cells were then resuspended in RPMI-1640 without phenol red (Life Technologies; catalogue #32404014) that was supplemented with 5 mM HEPES.

### TIRF microscopy

All single-molecule fluorescence microscopy was done at 37 °C with an Olympus TIRFM system based on an inverted microscope (Olympus IX81) equipped with a 150X NA 1.45 TIRFM objective (Olympus), motorized filter wheel (Olympus), high performance electron multiplier (EM)-charge-coupled device (CCD) camera (Photometrics Evolve), and real-time data acquisition software (Metamorph). The 405 nm or 561 nm solid-state diode lasers were used to excite Qdot- or Cy3-labelled samples. Simultaneous two-channel recording was accomplished using a DC2 emission splitting system (Olympus) and a second EMCCD camera (Photometrics Evolve). Labelled B cells were pre-warmed to 37 °C for 5 min and then added to functionalized coverslips in a Chamlide magnetic chamber (Quorum Technologies; catalogue #CM-B18-1) that was assembled into a top-stage incubation system. The TIRF plane was adjusted to yield a penetration depth of ~85–90 nm from the coverslip and TIRF imaging was carried out for 10 s at 33 Hz. For wide-field imaging (in Fig. S9), SPT was first done in TIRF mode (as described above) and immediately switched to widefield mode and labelled receptors on the dorsal surface of cells were focused on prior to imaging.

### Particle detection and tracking

Single particles were detected using Icy bioimaging analysis software^64^. Particle detection was done using the UnDecimated Wavelet Transform Detector plugin, which was set to detect bright spots over a dark background, exhibit 50% sensitivity to spots <3 pixels, and apply size filtration in order to detect objects ranging from 2 pixels to 3000 pixels. Tracking parameters were set to recognize particles that exhibited either diffusive or directed motion. Particle tracking was done using the Multiple Hypothesis Tracking method built into the spot tracking plugin of Icy^24, 64, 65^. Output files indicating the time and x-y positions of each particle were subjected to the mathematical analyses described below.

### Immobility threshold

Qdots, Cy3-labelled anti-IgM Fabs, and TetraSpeck beads (100 nm diameter; Thermo Fisher, catalogue #T7279) were diluted in MilliQ water, plated onto clean coverslips, and dried to obtain a dispersed layer of individually stuck particles. The coverslips were gently washed to remove any free-floating particles and the stuck particles were then imaged by TIRFM for 10 s at 33 Hz. Tracks generated from >300 adhered particles were analyzed to determine a distribution of their diameter, i.e. the maximum distance between any two points of the track. The 95^th^ percentile value of this distribution was set as the ‘immobility threshold’, which was used to remove effectively immobile particles from each set of trajectories prior to conducting all analysis.

### Particle Localization Precision

The apparent tracks of the stuck particles were also used to estimate the overall positional accuracy of our combined imaging, detection, and tracking systems. The standard deviation of the apparent particle displacements for these stuck particles estimates the positional precision, which is important when estimating diffusion coefficients (below). These were found to be 29 nm and 36 nm for Qdots and Fabs, respectively. Consistently, an identical experiment using an immobilized 100 nm TetraSpeck bead gave a positional accuracy of 14 nm.

To understand the importance of relative sources of error, we also assessed the detection accuracy of Icy’s spot detector. We simulated images of stationary particles with signal to noise ratios representative of our SPT data, performed spot detection using Icy, and then found the standard deviations of the displacement of ICY’s detected spots from the ground-truth simulated positions. To estimate the signal to noise ratio (SNR) of an image (a single frame of an SPT movie), we used the formula *SNR* = *I*/*σ*, where *I* is the signal intensity and *σ* is the standard deviation of the noise. To estimate *σ*, we found the median absolute deviation of pixel intensities in the image and multiplied this by 1.4826^66, 67^. To estimate *I*, we convolved the image with a 3 × 3 averaging filter (to smooth the noise) and took the maximum pixel intensity of the blurred image, minus the median pixel intensity of the original image. In the simulated images, the spots had Gaussian intensity profiles with widths chosen to match the expected point spread functions (PSFs) for our fluorescent probes and imaging system, and the noise was Gaussian white noise. For simulated Qdots, we found an error estimate of 23 nm (experimental SNR was ~47) whereas simulated Fab-Cy3 probes gave an error estimate of 30 nm (experimental SNR was ~7). We used these error estimates when estimating diffusion coefficients.

### Mean square displacement (MSD) plots

Simple MSD plots were constructed for each track by calculating the mean square displacement for a range of lag times according to the usual formula:

$$\langle {r}_{n}^{2}\rangle =\frac{1}{N-n}{\sum }_{i=1}^{N-n}\sqrt{{(x(i+n)-x(i))}^{2}+{(y(i+n)-y(i))}^{2}}.$$

Here, n is the lag time and N is the total time for a track. We then plotted the average MSD over all tracks for a given experimental condition against lag time.

### Single-state analysis of BCR trajectories

The single-state model assumes that displacements arise from a two-dimensional Brownian diffusion process. Diffusion coefficients (*D*) were calculated for individual tracks using a maximum likelihood estimation approach, exactly as described in Berglund^29^ taking into account noise due to positional errors in acquisition and blurring during acquisition. The measure of positional error in the method was fixed as described above. The implementation was tested using simulated trajectories.

### Two-state HMM analysis of BCR trajectories

We also analyzed BCR tracks using a two-state HMM, as described previously^33^. Briefly, this algorithm assumes that individual trajectories dynamically switch from rapidly diffusing (free) to slowly diffusing (apparent confinement) behaviours. We apply a likelihood maximization scheme to compute the distribution of diffusion coefficients for each state, as well as the probability of transitions between the two states that best match the observed trajectories. Per-frame transition probabilities were converted to first-order transition rates by multiplying them by the frame rate. The long-term time-averaged diffusivity D_eff_ is calculated by (D_slow_ K_fast→slow_ + D_fast_ K_slow→fast_)/(K_fast→slow_ + K_slow→fast_) and K_eff_ (the effective affinity of the transition to the slow state) is given by K_slow→fast_ divided by K_fast→slow_.

### Confinement analysis using a first-passage time (FPT) algorithm

To assess track confinement we applied the method described by Rajani *et al*.^43^, which was based on an approach developed by Fauchald and Tveraa^68^. In this context, first-passage time refers to the time required for a diffusing particle to escape a circle of radius *r* that is centered at its current position. Within each track, the distribution of times required to escape from that radius can be calculated for all points of the track. The variance of this distribution measures the amount of heterogeneity at the spatial scale of *r* ^43^. By plotting this variance against *r*, one can determine the spatial scale that exhibits the most heterogeneity. This radius, corresponding to the maximum variance, is then interpreted as an effective confinement radius for the track. It is important to note that this analysis is not independent of track duration. As shown with simulated data in Supplemental Fig. S7, a particle must have enough time to explore its confinement zone and meet the confining barrier several times in order for the confinement radius to be properly estimated. In order to properly compare sets of tracks from different experimental conditions, we performed this analysis on experimental tracks containing 50 frames (many short tracks) and 300 frames (fewer long tracks) and generated plots of the average over all such tracks.

### Data and algorithm availability

Raw imaging data (movies) and all processed particle tracks are available from the corresponding author (D.C.) on reasonable request. On request we will also provide our user-friendly MATLAB-based graphical user interface (GUI) called SPT-TIRF Track Analyzer for determining diffusion coefficients and carrying out FPT analysis of receptor confinement.

## Electronic supplementary material

Supplementary Information

Supplemental Video 1

Supplemental Video 2
