# Supplementary material for: Limitations of Qdot labelling compared to directly-conjugated probes for single particle tracking of B cell receptor mobility
Source: Sci Rep. 2017 Sep 12;7:11379. doi: 10.1038/s41598-017-11563-9 (PMC5595841; doi:10.1038/s41598-017-11563-9)
Supplement: Supplementary file 1 — Supplementary Information [file 41598_2017_11563_MOESM1_ESM.pdf]

Supplementary Information for:

Limitations of Qdot labelling compared to directly-conjugated probes for single particle tracking of B cell receptor mobility

Libin Abraham[1,2,3], Henry Y Lu[1,2,*], Rebeca Cardim Falcão[3,*], Joshua Scurll[2,3], Timothy Jou[1], Brian Irwin[3], Reza Tafteh[4], Michael R Gold[1,2†] and Daniel Coombs[3†]

[1]Department of Microbiology & Immunology, University of British Columbia, 2350 Health Sciences Mall, Vancouver, British Columbia, Canada V6T 1Z3
[2]Life Sciences Institute I[3] and Cell Research Groups, University of British Columbia, 2350 Health Sciences Mall, Vancouver, British Columbia, Canada V6T 1Z3
[3]Department of Mathematics and Institute of Applied Mathematics, 1984 Mathematics Road, University of British Columbia, Vancouver, British Columbia, Canada V6T 1Z2
[4]Department of Chemistry, University of British Columbia, Vancouver, British Columbia, Canada V6T 1Z1

[*] Authors contributed equally
[†] Corresponding authors

Contact:
coombs@math.ubc.ca
mgold@mail.ubc.ca

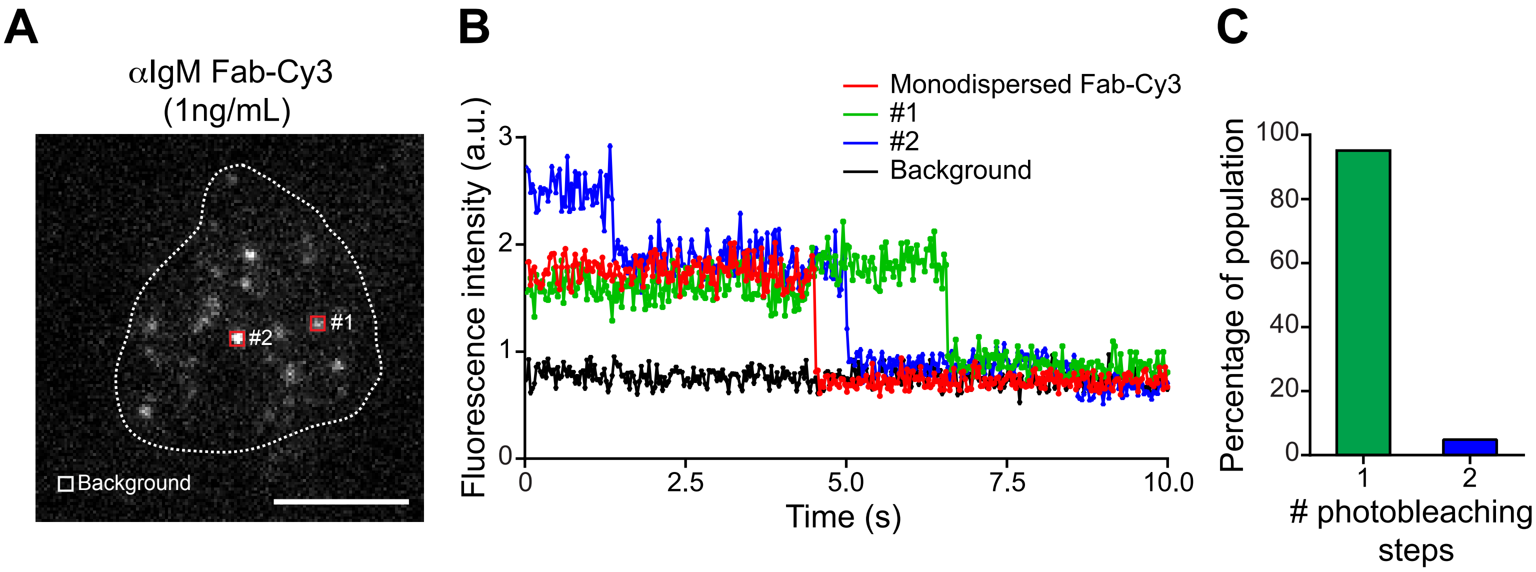
A
αIgM Fab-Cy3
(1ng/mL)
#2
#1
Background
B
Monodispersed Fab-Cy3
#1
#2
Background
Fluorescence intensity (a.u.)
3
2
1
0
0
2.5
5.0
7.5
10.0
Time (s)
C
Percentage of population
100
80
60
40
20
0
1
2
photobleaching steps

Figure S1. **Single-particle labeling of IgM BCRs on the B cell surface**

Validation of single particle labeling was conducted as previously described [8,41]. Briefly, *ex vivo* splenic B cells were labelled on ice with limiting dilution (1 ng/mL) of anti-IgM Fab-Cy3 and then allowed to settle onto anti-MHCII-coated coverslips for 5 min at 4°C. The cells were then fixed with 4% paraformaldehyde plus 0.2% gluteraldehyde for 90 min at 4°C to immobilize the BCRs. Samples were imaged by TIRF microscopy at 33 Hz for 10 s with laser settings that allowed photobleaching to be observed. A still image from a representative video is shown in the A. The white dashed line indicates the cell boundary. Scale bar = 5 μm. (B) Fluorescence intensities in the indicated regions were compared to that measured for monodispersed soluble anti-IgM Fab-Cy3 imaged using the same settings. Single-particle labelling is indicated by a single quantized fluorescence decrease (e.g. region #1, green trace) of similar magnitude to that of the monodispersed anti-IgM Fab-Cy3 (red trace). The black trace represents the background fluorescence intensity, monitored over time, in a region devoid of cells (indicated on the image). Although the majority of spots contained a single Cy3-labelled Fab, some contained two, e.g. region #2 (blue trace). (C) This photobleaching analysis was performed on >230 random particles and the percentage of particles that had single-step and two-step photobleaching is indicated. Approximately 95% of the spots exhibited single-step photobleaching.

Abraham *et al.*, Supplemental Fig. 2
(Refers to Fig. 1)

A

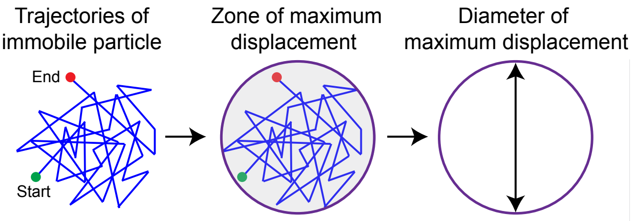

Sample	# Tracks	Diameter of maximum displacement (nm)			
		Mean	Median	90%	95%
Tetraspek bead	911	74.85	65.88	104.14	117.01
Q dot	278	115.75	106.60	152.83	179.55
Fab-Cy3	502	147.16	134.41	208.14	234.33

B

Cell Type	Receptor	Trial #	Label	# Tracks	# Excluded	% Excluded
Splenic B cells	IgM	1	Fab-Cy3	2481	7	0.282
			Fab-biotin-Qdot	1775	39	2.198
		2	Fab-Cy3	2926	0	0
			Fab-biotin-Qdot	2091	33	1.578
		3	Fab-Cy3	3489	1	0.029
			Fab-biotin-Qdot	1528	29	1.898
A20 B lymphoma	IgG	1	Fab-Cy3	2212	14	0.633
			Fab-biotin-Qdot	2769	25	0.903
		2	Fab-Cy3	1851	1	0.054
			Fab-biotin-Qdot	1599	22	1.376
		3	Fab-Cy3	2132	5	0.235
			Fab-biotin-Qdot	4249	81	1.907

Figure S2: Immobility threshold of stuck fluorophores

(A) Schematic representation showing the diameter of maximum displacement for a stuck particle (*upper panel*). The immobility thresholds for Tetraspek beads as well as Fab-Cy3 and Qdot fluorescent probes were determined as described in the methods. Values for the median, mean, 90th, and 95th percentile confinement diameters are shown for each type of stuck particle (*lower panel*). (B) The total number of tracks obtained in each experiment is shown along with the number of tracks removed after applying their respective immobility thresholds and the percentage of tracks removed. For Fab-Cy3 probes less than 1% of tracks were removed. For the less mobile Qdots, the percent of tracks that were removed by applying the immobility threshold never exceeded 2.3%.

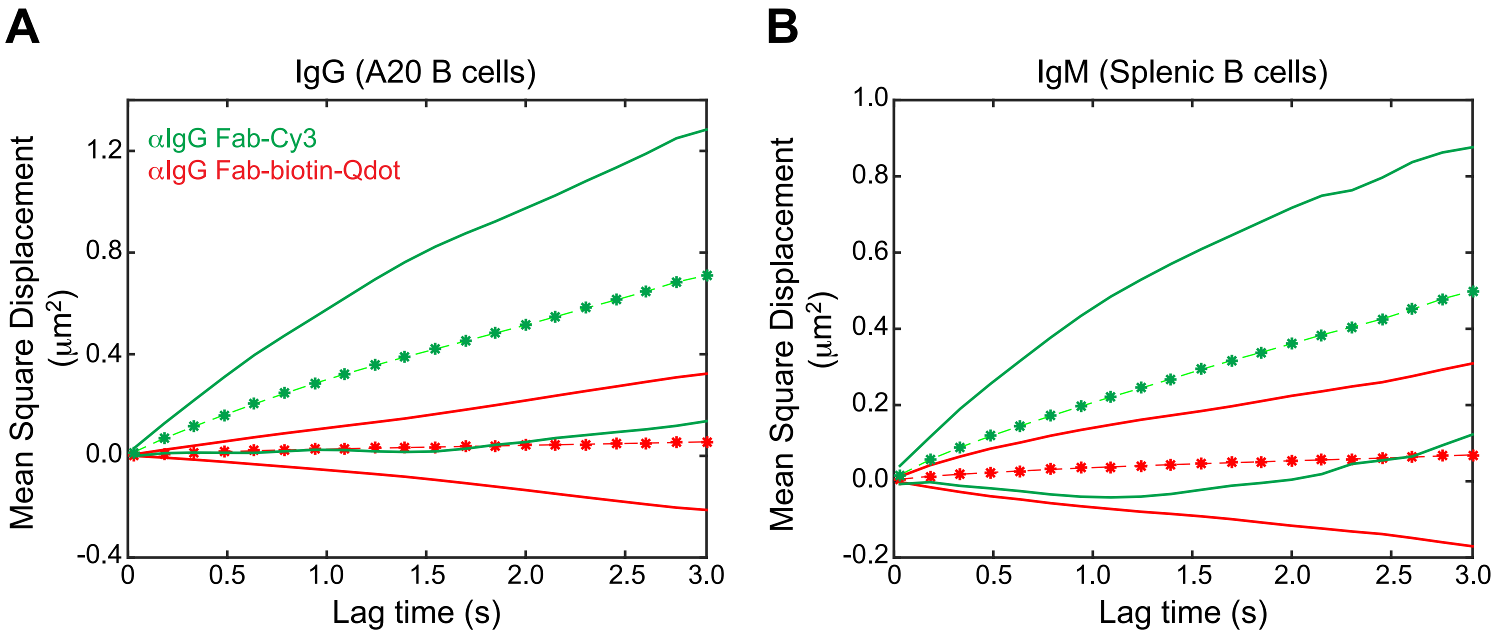
A
IgG (A20 B cells)
αIgG Fab-Cy3
αIgG Fab-biotin-Qdot
Mean Square Displacement (μm²)
Lag time (s)
1.2
0.8
0.4
0.0
-0.4
0
0.5
1.0
1.5
2.0
2.5
3.0
B
IgM (Splenic B cells)
Mean Square Displacement (μm²)
Lag time (s)
1.0
0.8
0.6
0.4
0.2
0.0
-0.2

Figure S3: Mean square displacement (MSD)

MSD of the tracks were calculated as described in methods. Indicated are MSD plots of IgG labelled receptors (A) in A20 B cells and IgM labelled receptors (B) in *ex vivo* primary B cells, corresponding to Figure 1 in the main text. The lag time ranged from 1 to 100 frames (0.030 s to 3.3 s). Error bars show the standard error of the mean of the MSD at that point.

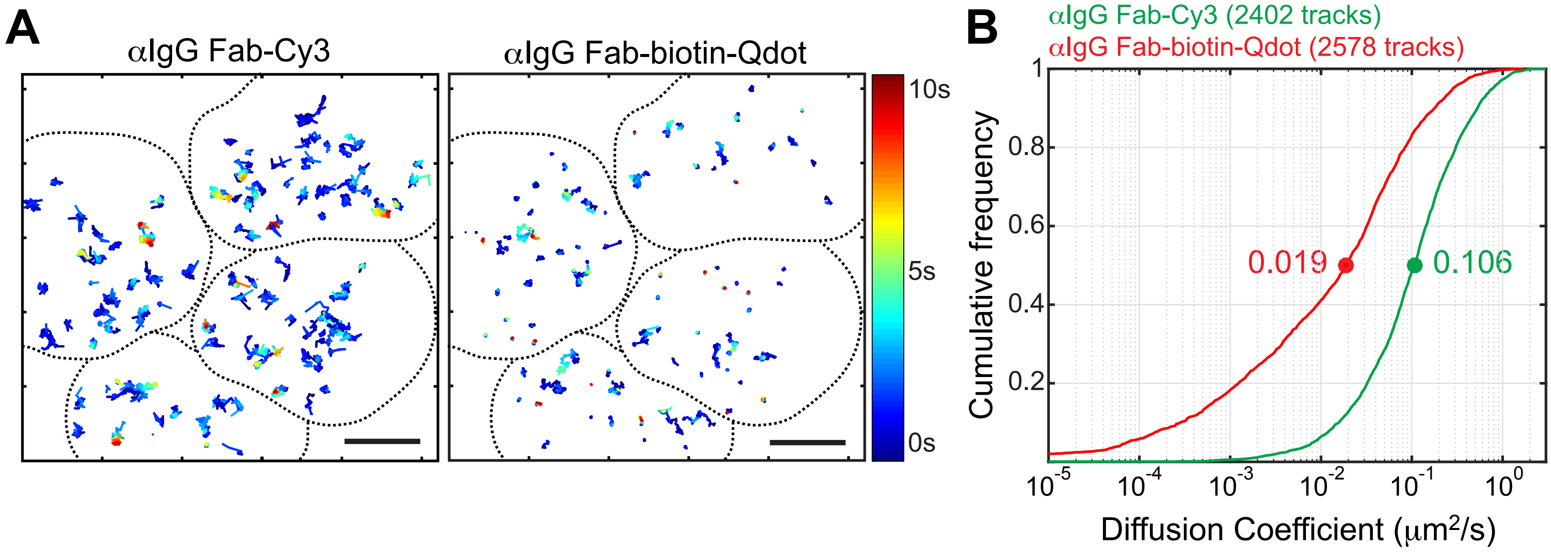
A
αIgG Fab-Cy3
αIgG Fab-biotin-Qdot
10s
5s
0s
B
αIgG Fab-Cy3 (2402 tracks)
αIgG Fab-biotin-Qdot (2578 tracks)
Cumulative frequency
1
0.8
0.6
0.4
0.2
0.019
0.106
10^{-5} 10^{-4} 10^{-3} 10^{-2} 10^{-1} 10^{0}
Diffusion Coefficient ($\mu m^2/s$)

Figure S4: Dual color imaging of IgM BCRs using Fab-Cy3 and Fab-biotin-Qdot

(A) IgM BCRs on *ex vivo* B cells were labelled with both anti-IgM Fab-Cy3 and anti-IgM Fab-biotin-525 nm Qdots. Two-colour SPT using two EMCCD cameras were performed in order to simultaneously image Cy3- and Qdot-labelled BCRs on the same cell, as described in Methods. Trajectories are plotted based using a colour-coded temporal scale. The dashed lines indicate the boundary of the cell. Scale bars = 3 μm. (B) Cumulative frequency curves of diffusion coefficients were generated separately for IgM-BCRs labelled with anti-IgM Fab-Cy3 versus anti-IgM Fab-biotin-Qdot. Median values are indicated by the dots on the curves.

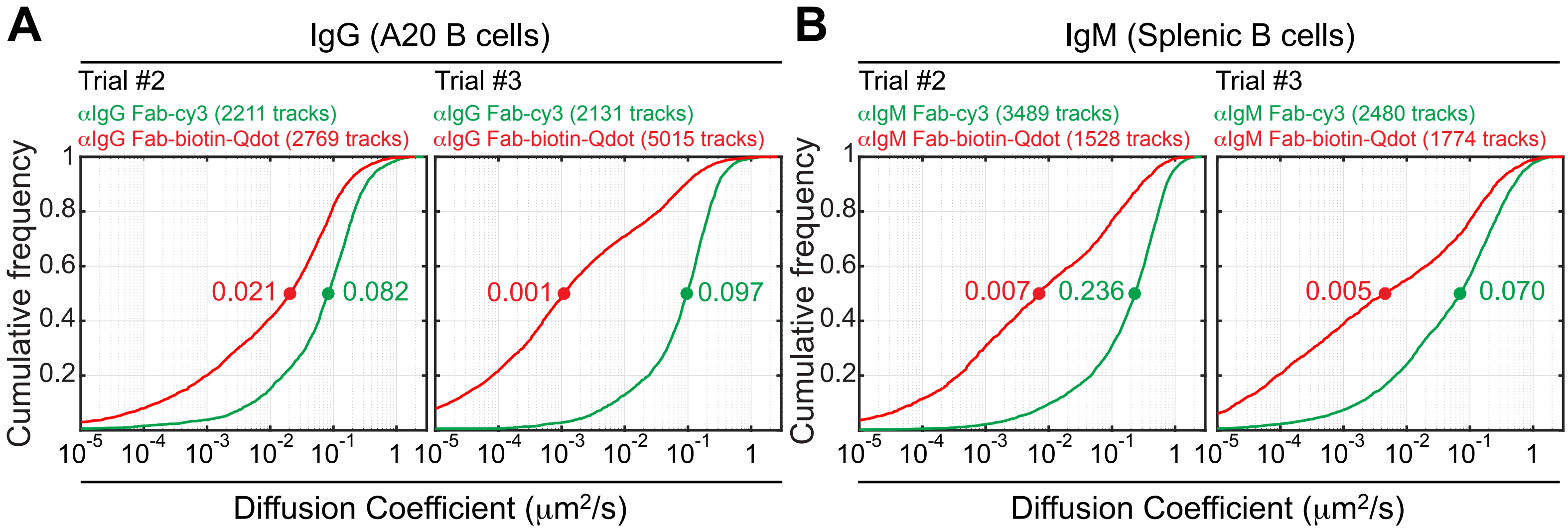
A
IgG (A20 B cells)
Trial #2
αIgG Fab-cy3 (2211 tracks)
αIgG Fab-biotin-Qdot (2769 tracks)
0.021
0.082
Trial #3
αIgG Fab-cy3 (2131 tracks)
αIgG Fab-biotin-Qdot (5015 tracks)
0.001
0.097
Cumulative frequency
Diffusion Coefficient (μm²/s)
B
IgM (Splenic B cells)
Trial #2
αIgM Fab-cy3 (3489 tracks)
αIgM Fab-biotin-Qdot (1528 tracks)
0.007
0.236
Trial #3
αIgM Fab-cy3 (2480 tracks)
αIgM Fab-biotin-Qdot (1774 tracks)
0.005
0.070
Cumulative frequency
Diffusion Coefficient (μm²/s)

Figure S5: Multiple independent experiments show that Qdots hinder the lateral mobility of cell surface receptors

(A-B) IgG-BCRs on A20 B cells (A) or IgM-BCRs on *ex vivo* murine splenic B cells (B) were labelled with Fab-Cy3 or Fab-biotin-Qdot probes and imaged by TIRF microscopy as in Fig. 1. Depicted are data from two additional independent trials of the same experiments shown in Fig. 1B-D (A20 B cells) or Fig. 1E-G (primary B cells). Cumulative frequency curves of diffusion coefficients for individual BCR tracks are shown. Median values are indicated by the dots on the curves.

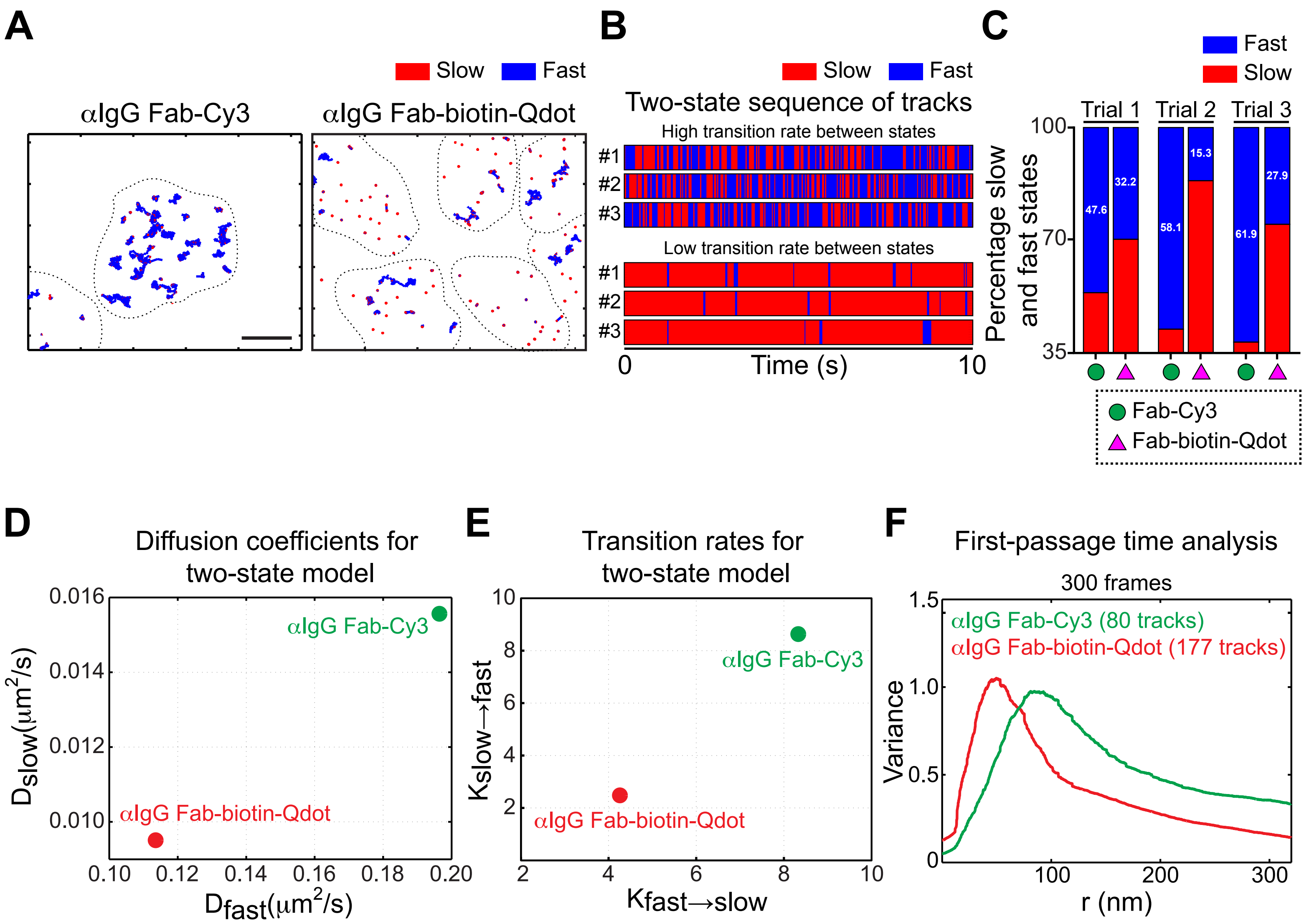
A
Slow Fast
αIgG Fab-Cy3
αIgG Fab-biotin-Qdot
B
Slow Fast
Two-state sequence of tracks
High transition rate between states
#1
#2
#3
Low transition rate between states
#1
#2
#3
0
Time (s)
10
C
Fast
Slow
Trial 1
Trial 2
Trial 3
Percentage slow and fast states
100
70
35
47.6
32.2
58.1
15.3
61.9
27.9
Fab-Cy3
Fab-biotin-Qdot
D
Diffusion coefficients for two-state model
αIgG Fab-Cy3
αIgG Fab-biotin-Qdot
$D_{slow}(\mu m^2/s)$
$D_{fast}(\mu m^2/s)$
0.016
0.014
0.012
0.010
0.10
0.12
0.14
0.16
0.18
0.20
E
Transition rates for two-state model
αIgG Fab-Cy3
αIgG Fab-biotin-Qdot
Kslow→fast
Kfast→slow
10
8
6
4
2
2
4
6
8
10
F
First-passage time analysis
300 frames
αIgG Fab-Cy3 (80 tracks)
αIgG Fab-biotin-Qdot (177 tracks)
Variance
r (nm)
1.5
1.0
0.5
0
100
200
300

Figure S6: Qdot labelling alters state-switching behaviour and apparent confinement of IgG BCRs

(A) A20 B-lymphoma cells were labelled with anti-IgG Fab-Cy3 or anti-IgG-biotin-Qdot. After removing applying the immobility threshold to remove stuck particles, the two-state HMM algorithm was used to subdivide trajectories into slow-diffusing (red) and fast-diffusing (blue) segments with dynamic transitions. Representative static trajectories of IgG-BCRs that were segmented into inferred slow and fast states. Scale bar = 5 µm. (B) Each barcode shows the time course for transitions between fast (blue) and slow (red) states. Shown are 3 examples of trajectories in which the receptor rapidly switches between slow and fast states (these were obtained using anti-IgG Fab-Cy3 labelling) with a high transition rate and 3 trajectories in which the receptor exhibits primarily slow diffusion, with a low transition rate (these were obtained using anti-IgG Fab-biotin-Qdot labelling) (C) All tracks for a given condition were combined and the percent of time that receptors exhibited slow (red) versus fast (blue) diffusion was determined. Data are shown for 3 independent experiments. (D-E) The HMM algorithm was then used to calculate the diffusion coefficients for the slow and fast states (D), as well as the transition rates ($K_{slow \rightarrow fast}$, $K_{fast \rightarrow slow}$) (E) between the two states. In panel F, the trajectories were analyzed using the FPT algorithm and confinement radius histograms are shown for long tracks (300 frames).

Abraham *et al.*, Supplemental Fig. 7
(Refers to Fig. 3)

A

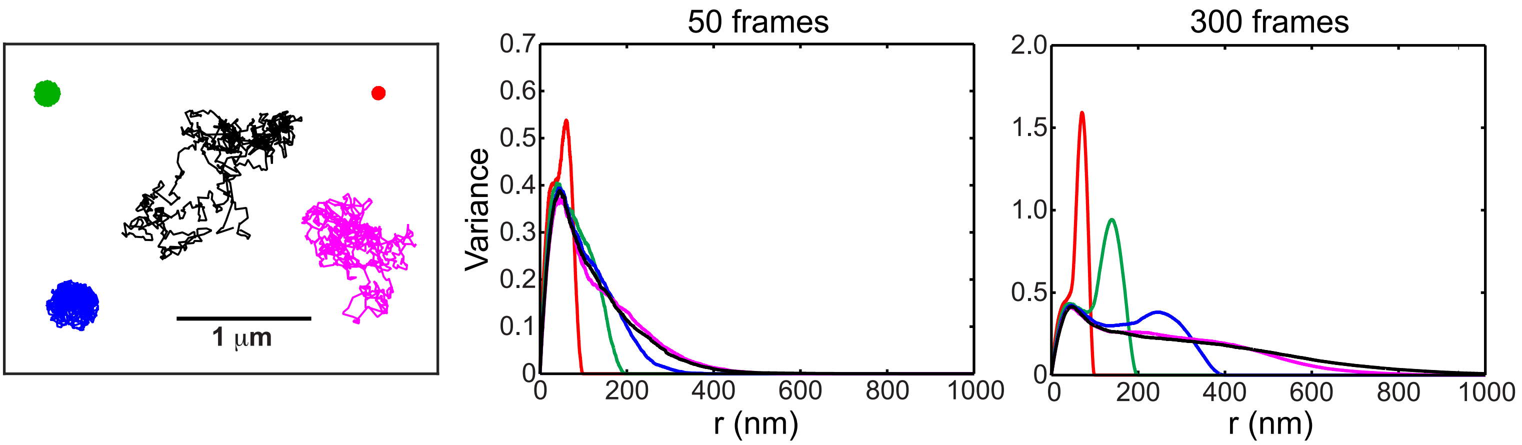

B

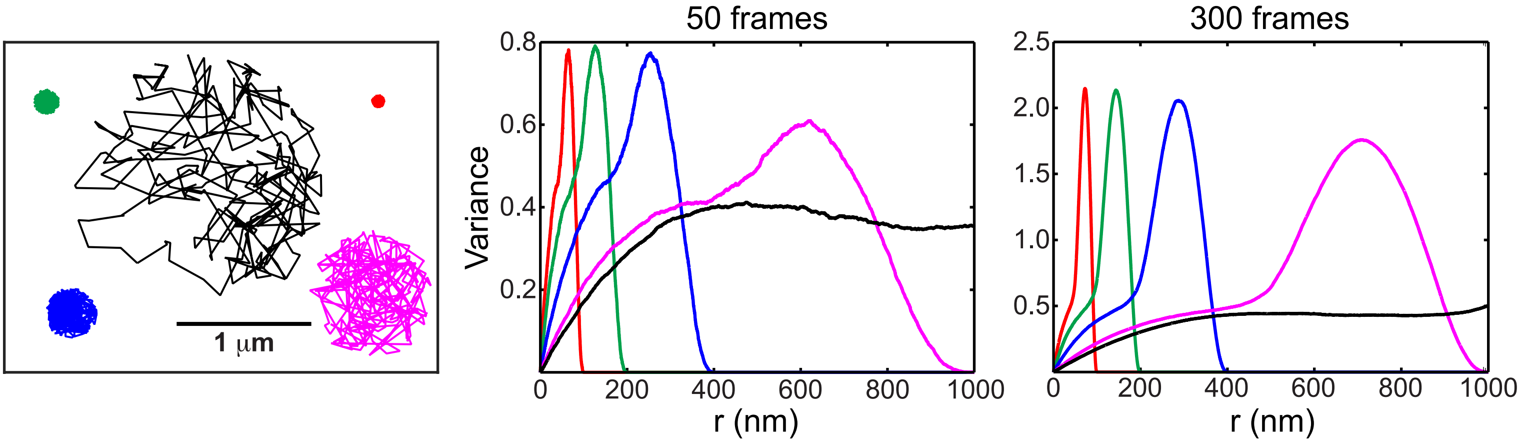

Figure S7: Validation and optimization of First Passage Time (FPT) algorithm

(A-B) Simulated trajectory of particles undergoing Brownian diffusion that is confined to circular domains radii of 50 nm (red), 100 nm (green), 200 nm (blue), 500 nm (magenta), or 1000 nm (black) (*left panels*). All particles were assumed to have a diffusion coefficient of D = $5x10^{-9}$ $\mu m^2/s$. In (A) the particles were assumed to have been detected continuously in all 1000 frames of a 1 second-long video with a frame rate of 1000 Hz. In (B) the particles were assumed to have been detected continuously in all 330 frames of a 10 second-long video with a frame rate of 33 Hz (similar to our experimental data). FPT analyses were performed, and the variance was calculated for 10 trajectories for each defined confinement radius. Graphs are shown for short tracks (*middle panels*) and long tracks (*right panels*).

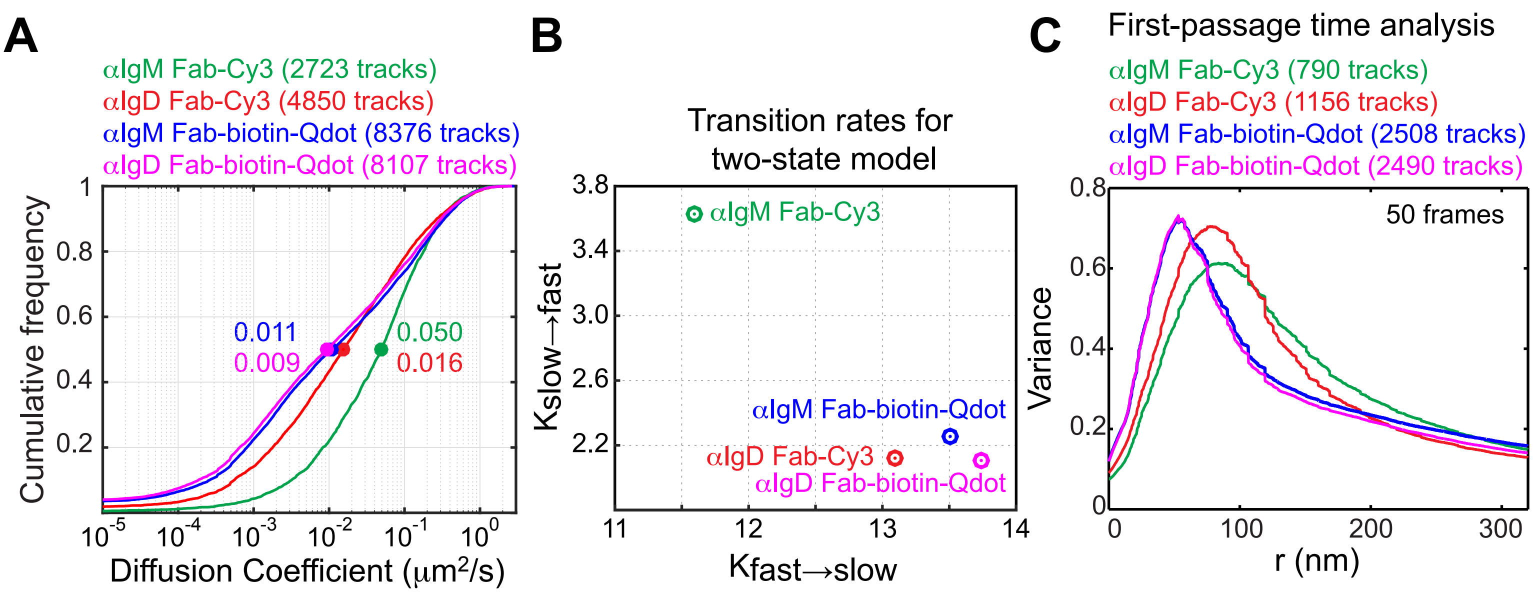
A
αIgM Fab-Cy3 (2723 tracks)
αIgD Fab-Cy3 (4850 tracks)
αIgM Fab-biotin-Qdot (8376 tracks)
αIgD Fab-biotin-Qdot (8107 tracks)
Cumulative frequency
0.011
0.009
0.050
0.016
Diffusion Coefficient (μm²/s)
B
Transition rates for two-state model
αIgM Fab-Cy3
αIgM Fab-biotin-Qdot
αIgD Fab-Cy3
αIgD Fab-biotin-Qdot
Kslow→fast
Kfast→slow
C
First-passage time analysis
αIgM Fab-Cy3 (790 tracks)
αIgD Fab-Cy3 (1156 tracks)
αIgM Fab-biotin-Qdot (2508 tracks)
αIgD Fab-biotin-Qdot (2490 tracks)
50 frames
Variance
r (nm)

Figure S8: Lateral diffusion of IgM-BCRs and IgD-BCRs cannot be distinguished using Qdot labelling.

Ex vivo splenic B cells were labelled with anti-IgM Fab-Cy3 or anti-IgM Fab-biotin-Qdot. Panel (A) shows cumulative frequency plots of the diffusion coefficients for all tracks. Median values are indicated by the dots. (B) Tracks were analyzed using the two-state HMM and the inferred transition rates ($K_{slow \rightarrow fast}$, $K_{fast \rightarrow slow}$) between the two states are shown. (C) FPT analysis indicating the confinement radii of the tracks.

Abraham *et al.*, Supplemental Fig. 9
(Refers to Fig. 5)

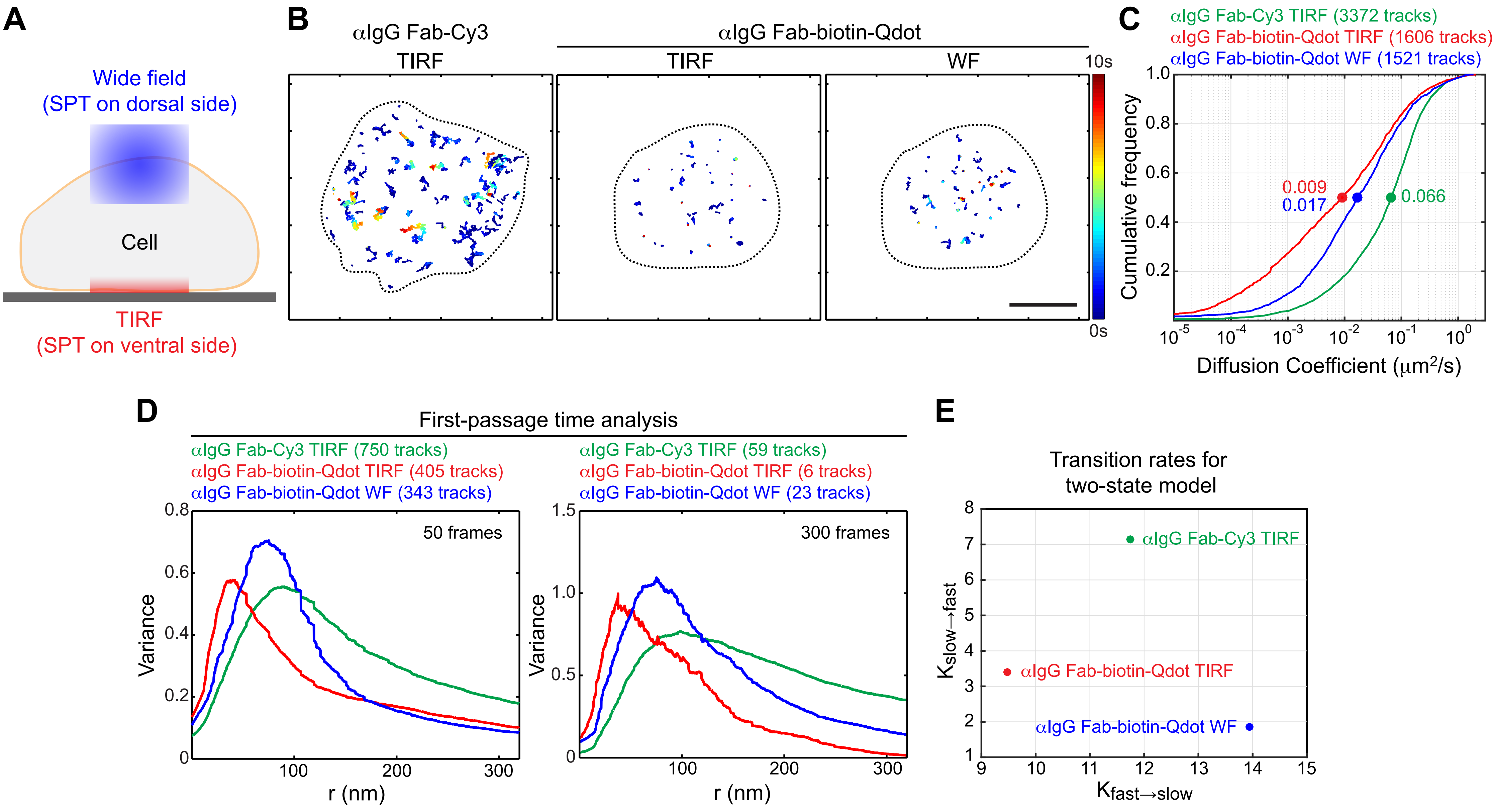

Figure S9: Qdot labelling impair receptor diffusion in both TIRF and wide-field
(A) Schematic representation of imaging modes. IgG BCRs on A20 B cells were labelled with either anti-IgG Fab-Cy3 or anti-IgG Fab-biotin-Qdot and settled on anti-MHC II coated coverslips before tracking. Fab-Cy3 labelled receptors were imaged in TIRF mode at a penetration depth of ~ 90 nm (shown in red) to visualize receptors on ventral side of cell, at the membrane-coverslip interface. Qdot labelled receptors were imaged first in TIRF and immediately switched to widefield mode by focussing on receptors present on the dorsal side of the cell (indicated in blue). (B) Static trajectories are plotted using a colour-coded temporal scale. The dashed lines indicate cell boundaries. Scale bars = 3 μm. (C) Cumulative frequency plots of diffusion coefficients. Median values are indicated by the dots on the curves. In panel D, tracks were analyzed using the FPT algorithm. Histograms depicting the confinement radii for short tracks (50 frames, *left panel*) and long tracks (300 frames, *right panel*) are shown. (E) Tracks were analyzed using the two-state HMM and the transition rates ($K_{slow \rightarrow fast}$, $K_{fast \rightarrow slow}$) between the two states are depicted.

Supplemental Table 1 (Refers to Fig. 1 and S3)

Receptor	Trial #	Label	# Tracks	D_{slow} ($10^{-3}\mu m^2/s$)	D_{fast} ($10^{-3}\mu m^2/s$)	Deff	$K_{slow\rightarrow fast}$ (s^{-1})	$K_{fast\rightarrow slow}$ (s^{-1})	Keff = ($K_{slow\rightarrow fast}/K_{fast\rightarrow slow}$)
**IgM**	1	Fab-Cy3	2926	**35.68** [35.10-36.25]	**468.90** [460.76-477.04]	**170.37**	**5.61** [5.47-5.74]	**12.42** [12.04-12.81]	**0.45**
		Fab-biotin-Qdot	2091	**10.56** [10.46-10.66]	**236.07** [231.69-240.46]	**50.55**	**1.88** [1.83-1.93]	**8.73** [8.42-9.05]	**0.22**
	2	Fab-Cy3	3489	**28.90** [28.37-29.43]	**446.39** [439.94-452.85]	**190.42**	**6.73** [6.60-6.86]	**10.66** [10.42-10.91]	**0.63**
		Fab-biotin-Qdot	1528	**8.73** [8.62-8.84]	**190.12** [186.12-194.12]	**44.31**	**1.69** [1.63-1.76]	**6.94** [6.59-7.29]	**0.24**
	3	Fab-Cy3	2481	**20.23** [19.92-20.53]	**293.40** [288.33-298.47]	**101.09**	**3.48** [3.39-3.58]	**8.29** [8.03-8.54]	**0.42**
		Fab-biotin-Qdot	1775	**6.64** [6.58-6.70]	**183.04** [180.20-185.87]	**43.9**	**1.49** [1.45-1.53]	**5.56** [5.34-5.78]	**0.27**
**IgG**	1	Fab-Cy3	2212	**15.39** [14.84-15.94]	**197.99** [194.31-20.17]	**102.25**	**8.91** [8.64-9.19]	**9.82** [9.54-10.11]	**0.91**
		Fab-biotin-Qdot	2769	**9.52** [9.40-9.64]	**113.79** [112.13-115.45]	**43.07**	**2.98** [2.89-3.06]	**6.27** [6.03-6.51]	**0.48**
	2	Fab-Cy3	2132	**10.55** [9.92-11.17]	**174.27** [171.02-177.51]	**107.09**	**13.36** [13.03-13.70]	**9.63** [8.97-9.30]	**1.39**
		Fab-biotin-Qdot	4249	**8.45** [8.40-8.51]	**113.02** [111.39-114.66]	**24.5**	**1.2** [1.17-1.24]	**6.64** [6.43-6.86]	**0.18**
	3	Fab-Cy3	1851	**9.98** [9.24-10.73]	**204.45** [200.43-208.52]	**130.31**	**14.81** [14.42-15.21]	**9.13** [8.82-9.44]	**1.62**
		Fab-biotin-Qdot	1599	**9.18** [9.04-9.32]	**77.60** [76.09-79.12]	**28.26**	**2.75** [2.65-2.85]	**7.11** [6.84-7.37]	**0.39**

Supplemental Table 2, (Refers to Fig. 4,5)

Receptor	Label	Treatment	# Tracks	D_{slow} ($10^{-3}\mu m^2/s$)	D_{fast} ($10^{-3}\mu m^2/s$)	Deff	$K_{slow\rightarrow fast}$ (s^{-1})	$K_{fast\rightarrow slow}$ (s^{-1})	Keff = ($K_{slow\rightarrow fast}/K_{fast\rightarrow slow}$)
**IgM**	Fab-Cy3	none	2711	**28.26** [27.82-28.81]	**383.27** [375.54-391.01]	**125.87**	**4.86** [4.72-5.00]	**12.82** [12.17-12.82]	**0.3791**
**IgD**	Fab-Cy3	none	4972	**18.91** [18.76-19.04]	**278.05** [274.43-281.68]	**73.14**	**1.96** [1.91-2.01]	**7.4** [7.21-7.59]	**0.2649**
**IgM**	Fab-biotin-Qdot	none	1581	**10.29** [10.19-10.39]	**355.81** [348.88-362.74]	**68.49**	**2.04** [1.99-2.09]	**10.06** [9.82-10.30]	**0.2028**
**IgD**	Fab-biotin-Qdot	none	2663	**11.54** [11.41-11.66]	**265.83** [260.84-270.82]	**56.43**	**2.20** [2.14-2.26]	**10.26** [9.95-10.57]	**0.2144**
**IgM**	Fab-Cy3	DMSO	3738	**25.64** [25.33-25.95]	**338.49** [333.67-343.30]	**126.65**	**4.60** [4.50-4.70]	**9.64** [9.30-9.97]	**0.4772**
	Fab-Cy3	LatA	3528	**23.77** [23.10-24.44]	**414.65** [408.60-420.70]	**219.66**	**9.2** [8.91-9.39]	**9.16** [9.01-9.42]	**1.004**
	Fab-biotin-Qdot	DMSO	8442	**14.16** [14.09-14.24]	**506.87** [500.86-512.88]	**84.82**	**2.32** [2.29-2.36]	**13.87** [13.45-14.28]	**0.1673**
	Fab-biotin-Qdot	LatA	8573	**13.81** [13.73-13.90]	**420.85** [416.22-425.49]	**92.01**	**2.91** [2.87-2.95]	**12.23** [11.91-12.56]	**0.2379**
**IgM**	Fab-Cy3	BAFF	3875	**29.40** [29.02-29.78]	**387.78** [380.53-395.03]	**109.42**	**4.01** [4.00-4.19]	**14.24** [13.90-14.58]	**0.2816**
	Fab-Cy3	LPS	4834	**23.48** [23.00-23.97]	**344.57** [340.39-348.75]	**174.1**	**8.34** [8.17-8.50]	**9.44** [9.21-9.67]	**0.8835**
	Fab-biotin-Qdot	BAFF	1540	**10.23** [10.13-10.32]	**354.69** [347.92-361.45]	**68.18**	**2.04** [1.99-2.09]	**10.09** [9.86-10.32]	**0.2021**
	Fab-biotin-Qdot	LPS	3929	**10.80** [10.71-10.88]	**391.48** [386.75-39.22]	**101.93**	**2.86** [2.81-2.91]	**9.09** [8.86-9.31]	**0.3146**

Supplemental Table 3, (Refers to Fig. S9)

Receptor	Label	Imaging mode	# Tracks	D_{slow} ($10^{-3}\mu m^2/s$)	D_{fast} ($10^{-3}\mu m^2/s$)	Deff	$K_{slow\rightarrow fast}$ (s^{-1})	$K_{fast\rightarrow slow}$ (s^{-1})	Keff = ($K_{slow\rightarrow fast}/K_{fast\rightarrow slow}$)
**IgG**	Fab-Cy3	TIRF	3373	**20.60** [20.09-21.10]	**219.09** [215.13-223.04]	**95.67**	**7.14** [6.96-7.33]	**11.74** [11.43-12.05]	**0.6082**
	Fab-biotin-Qdot	TIRF	1606	**7.09** [6.95-7.23]	**167.05** [162.79-171.30]	**49.26**	**3.39** [3.28-3.51]	**9.48** [9.07-9.89]	**0.3576**
	Fab-biotin-Qdot	Widefield	1522	**20.83** [20.46-21.20]	**351.43** [335.74-367.11]	**59.55**	**1.85** [1.74-1.96]	**13.94** [13.11-14.78]	**0.1327**

Supplementary Tables S1-S3: Two-state Hidden Markov Model (HMM) analysis of BCR diffusion

After applying an immobility threshold to remove stuck particles (see Methods and Supplementary Fig. 2), trajectories were analyzed using two-state HMM model. This model assumes that individual trajectories consist of slow and fast segments and calculates the distribution of diffusion coefficients for each state as well as the probability of transitions between the two states. Diffusion coefficients for the slow and fast states (D_{slow}, D_{fast}) are depicted along with the frequency of transitions between the two states ($K_{slow \rightarrow fast}$, $K_{fast \rightarrow slow}$). D_{eff} is calculated by $[(D_{slow}\ K_{fast \rightarrow slow} + D_{fast}\ K_{slow \rightarrow fast})/(K_{fast \rightarrow slow} + K_{slow \rightarrow fast})]$; higher D_{eff} indicates faster diffusion coefficient. K_{eff} (given by the ratio of $K_{slow \rightarrow fast}$ divided by $K_{fast \rightarrow slow}$) indicates whether the predominant mode of switching is to the fast state (higher value of K_{eff}) or to the slow state (lower value of K_{eff}).

Supplementary table S1: IgM-BCRs on *ex vivo* splenic B cells or IgG-BCRs on A20 were labelled with either Fab-Cy3 or Fab-biotin-Qdot and trajectories were analyzed using a two-state HMM algorithm. The data represent three independent trials of the experiments shown in Fig. 1 and S5. The median values are shown and the ranges in parentheses indicate 95% confidence intervals.

Supplementary table S2: Two-state HMM analysis of IgM-BCRs and IgD-BCRs labelled using either Fab-Cy3 or Fab-biotin-Qdot probes. The data represent experiments shown in Figs. 4 and 5. Median values are shown and the ranges in parentheses indicate 95% confidence intervals.

Supplementary table S3: A20 B cells were labelled with either anti-IgG Fab-Cy3 or anti-IgG Fab-biotin-Qdot. Qdot labelled cells were imaged both TIRF and widefield. And the trajectories were analyzed using a two-state HMM algorithm. The data represent experiments shown in S9. The median values are shown and the ranges in parentheses indicate 95% confidence intervals.

Supplementary Video 1:
IgG-containing BCRs on A20 B cells were labelled with either Fab-Cy3 or Fab-biotin-Qdot and imaged at 33 Hz for 10 s in TIRF. Trajectories are plotted in different colors.

Supplementary Video 2:
Monodispered TetraSpeck beads (100nm) were adhered to coverglass and stuck particles were imaged at 33 Hz for 10 s in TIRF. Trajectories indicate maximum displacement during 10s. Pixel size = 106nm.
